# From emotional map to design criteria: verification of the correlation between community green space form and emotional health of high-density urban residents

**DOI:** 10.3389/fpubh.2025.1617294

**Published:** 2025-07-15

**Authors:** Tianyi Zhang, Xukui Wang, Junping He

**Affiliations:** Faculty of Architecture and Urban Planning, Kunming University of Science and Technology, Kunming, Yunnan, China

**Keywords:** community-healing landscape, emotional health, green landscape design, population preference, uplifted emotions, relaxed emotions

## Abstract

**Introduction:**

Rising public health awareness, driven by economic growth, underscores emotional health as a critical determinant of overall well-being. High-density urban development in Hong Kong’s Central and Western District severely limits accessible green spaces, exacerbating urban stress. This study investigates how tailored community green space design can mitigate this stress and enhance mental well-being by positively influencing residents’ emotions.

**Methods:**

Using isovist analysis and Grasshopper simulation, an emotional map reflecting residents’ sentiments across the district was generated. User preferences for green space characteristics (richness, flower-to-grass ratio, connectivity, and shape) and their emotional impacts were quantitatively analyzed using 455 validated questionnaires.

**Results:**

Key findings include: (1) Landscape Preference: The public favors medium-density landscapes and regularly shaped green spaces. (2) Emotion Regulation: Higher flower proportions enhance exhilaration, while greater green coverage promotes relaxation. Curved layouts stimulate exhilaration, whereas rectangular shapes and moderate connectivity facilitate relaxation. (3) Demographic Variations: Young people prefer highly connected “exploration spaces,” while the older adults favor tranquil spaces with higher flower-to-grass ratios and low density. Women express more positive emotions in green spaces, while men typically exhibit neutral responses.

**Discussion:**

The study demonstrates that strategically designed green spaces—considering density, floral elements, geometry, and connectivity—can effectively regulate emotions and alleviate urban stress. Age- and gender-specific preferences highlight the need for inclusive design approaches. These findings provide actionable insights for therapeutic landscape interventions, promoting community well-being and informing sustainable urban development in high-density contexts.

## 1 Introduction

Under the backdrop of rapid economic development, public health demands are increasingly shifting toward physical and mental well-being. Residents’ health has become a core issue of concern across various fields in China. Notably, emotional health serves as an important predictor of overall well-being. Clinical studies have demonstrated that emotions can influence the human body through intermediate mechanisms such as neurohumoural regulation, the endocrine system, and the immune system, and long-term negative emotions may lead to pathological changes in tissues and organs (1). In contrast, individuals with stable emotions tend to exhibit higher levels of mental health, stronger self-control abilities, and a greater sense of well-being (2). From the perspective of the 2030 Agenda for Sustainable Development, fostering harmony between individuals and their environment is essential for achieving sustainable urban development (3). Consequently, the concept of healthy and sustainable cities has emerged, with the aim of reintegrating nature into urban environments to enhance both physical and mental health.

In recent decades, scientific research has focused on elucidating the underlying mechanisms through which the natural environment positively influences mental health. Studies indicate that people show a stronger preference for vegetated environments than for urban spaces without vegetation (4). Ulrich (5) proposed that natural environments can effectively enhance positive emotions, reduce negative emotions, and alleviate mental stress by stimulating parasympathetic nervous system activity (5). Furthermore, plant-rich natural environments not only inspire positive emotions and relieve stress but also facilitate the restoration of cognitive and behavioral capabilities (6). Research has also demonstrated that landscape elements can significantly improve the emotional well-being and cognitive function of sick children while reducing non-symptom-related indicators and promoting relaxation (7). Likewise, landscape elements have been shown to significantly enhance the mental health of college students (8). Additionally, the rehabilitation effects of green spaces vary significantly depending on their landscape features. For example, factors such as the type, richness, quantity, and spatial layout of green spaces in parks are closely associated with rehabilitation outcomes (9).

As a critical component of the built environment, urban green spaces (UGSs) have been shown to decrease the morbidity and mortality rates of cardiovascular and respiratory diseases (10) and reduce socioeconomic health inequalities (11). Studies have shown that the shape of community green spaces can reduce noncommunicable diseases in people (12) and that design elements such as natural shapes have a direct effect on people’s physical and mental health (13). A study of health data from residents surveyed in New Zealand revealed that, compared with those in areas with low proportions of green space, residents in areas with high proportions of green space generally had fewer mental health problems and a lower risk of cardiovascular diseases. Alcock I et al. (14) studied survey data from British households and reported that the mental health of families who moved from areas with low proportions of green spaces in cities to areas with high proportions of green spaces generally improved. By observing the emotional healing effect on subjects after watching horror movies, Van den Berg et al. (15) reported that UGS can improve people’s moods. Triguero M et al. (16) conducted interviews with residents in Catalonia, Spain, and on the basis of sample cross-sectional data, they concluded that UGSs positively affect the mental health of residents under different urbanization, economic status and gender conditions. M. Gola et al. found during the COVID-19 pandemic that brief exposures to nature, particularly during periods of high stress such as a pandemic, can positively influence the well-being and mental health of individuals, especially healthcare staff (17).

In terms of research methods, existing studies predominantly utilize photo-based assessment approaches. Specifically, respondents are asked to evaluate the aesthetic value or assign preference scores to photos featuring different landscape elements. These landscape elements are subsequently objectively quantified and subjected to correlation analysis, thereby systematically revealing the underlying relationships between landscape elements and landscape preferences (18). Among the various combinations of landscape elements, people’s landscape preferences play a crucial role in guiding landscape design (19). Moreover, landscape preferences not only reveal users’ aesthetic tendencies but also highlight the positive impact of landscapes on human attention recovery (20, 21).

Previous studies have focused predominantly on investigating the relationships between UGSs and human physical and mental health (22) or have been restricted to examining the impact of park green spaces on emotions and the influence of community green space ratios on residents’ emotions (23). Nevertheless, research regarding individuals’ preferences for different types of community green spaces and the effects of combinations of green spaces on emotions remains relatively limited. In light of this, the present study places people at the core, thoroughly accounting for variations in individual landscape preferences, with the aim of exploring how to customize the design of community green spaces based on residents’ needs. This approach seeks to effectively mitigate urban living pressures and promote mental well-being.

Based on the visual field analysis method, this study elucidates the spatial distribution patterns of urban residents’ emotions in selected areas of Hong Kong’s Central and Western Districts and pinpoints low-emotion zones. Subsequently, through a questionnaire survey conducted within these zones, the study systematically investigates the influence of urban community green spaces across four dimensions: green spaces richness, the flower-to-grass ratio, connectivity, and shape. Additionally, it examines residents’ preferences for various green space combinations. The study employed SPSS software to conduct quantitative analyses of the correlations among various factors, with an emphasis on elucidating the underlying mechanisms through which different types and proportions of green spaces impact the two core emotions of exhilaration and relaxation. This research not only aims to clarify the positive regulatory role of green space design on individual emotions at the micro level but also aims to provide a scientific foundation for constructing livable urban environments and enhancing residents’ quality of life and well-being at the macro level.

Furthermore, this research focuses on the coordinated development between population growth and living environments. By optimizing the design of community green spaces, therapeutic green landscapes tailored for residents across different age groups and social backgrounds can be developed. This approach not only facilitates the promotion of population health and improvement of the urban microclimate but also offers robust scientific support for achieving sustainable urban development and enhancing public well-being.

## 2 Materials and methods

### 2.1 Residents’ emotion map based on isovist

The Central and Western District, as a high-end residential area on Hong Kong Island, despite its advantageous natural environment and well-established community facilities, still encounters a range of challenges. Characterized by a dense concentration of high-rise buildings, the district exhibits high building density and extremely low urban green coverage. Given that urban residents’ emotions are significantly influenced by the built environment and green space resources, this study addresses these issues by constructing a 3D model of the Mid-Levels area in Hong Kong using OpenStreetMap data. Building outlines, road networks, and terrain elevations were extracted to develop an urban model. In Grasshopper, the Ladybug plugin was utilized to distribute points evenly along roads for generating isovists, extracting parameters such as visible area, line-of-sight obstruction rate, and line-of-sight depth. Subsequently, clustering analysis was performed to delineate areas with high and low emotion values. Statistical analysis relied on a classic Multiple Linear Regression (MLR) model (Equation 1) (24):


(1) $${\text{Emotion}}_{j}=\alpha_{1}\mathrm{V\alpha}r_{1}+\alpha_{2}\mathrm{V\alpha}r_{2}+\ldots +\alpha_{n}\mathrm{V\alpha}r_{n}+\gamma +\varepsilon$$

**Figure 1:**
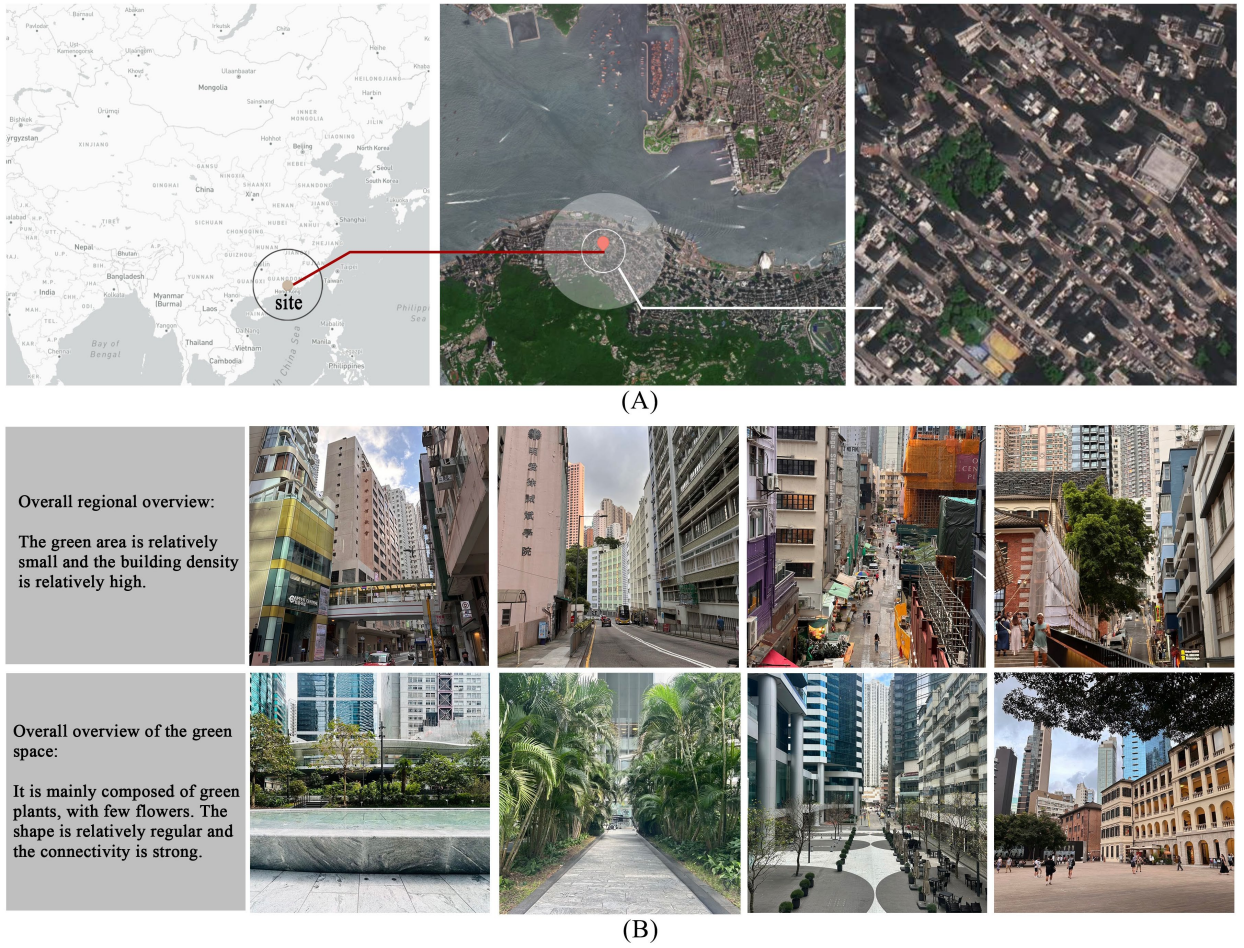
Overview of the research area. **(A)** Selection of research area locations; **(B)** Overview of green spaces.

Here, Emotion_j_ represents the average emotion value of all participants at aggregation point j. The model incorporates *n* visual exposure indicators as predictor variables. α_1_…α_n_ denote the coefficient estimates of the indicators Vαr_1_ through Vαr_n_ at aggregation point j. *γ* is the model intercept, and *ε* is the residual term. This formula was parameterized and integrated into Rhino for computational analysis, ultimately yielding the spatial distribution of emotions across the study area (Figure 1). The redder the color, the higher the emotional value, and the bluer the color, the lower the emotional value.

### 2.2 Online questionnaire design and procedure

**Figure 2:**
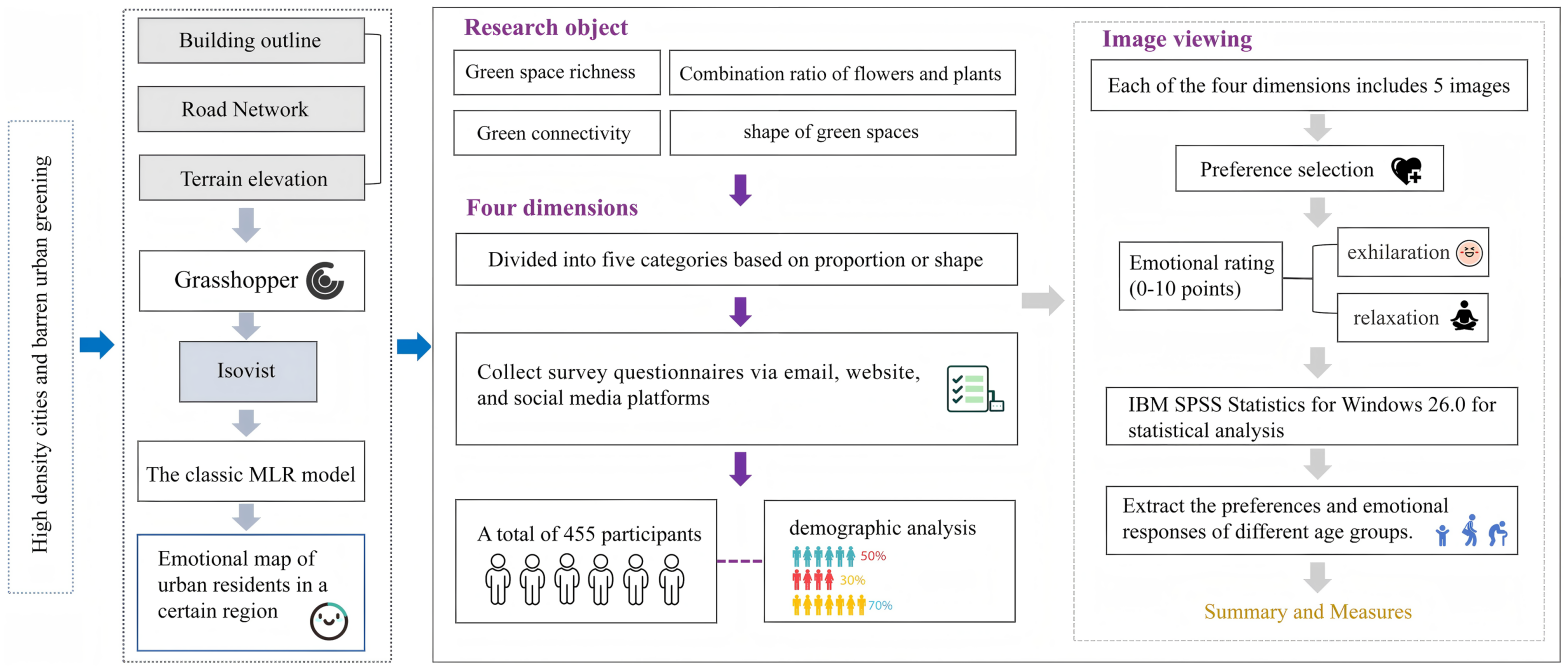
Research framework diagram.

**Figure 3:**
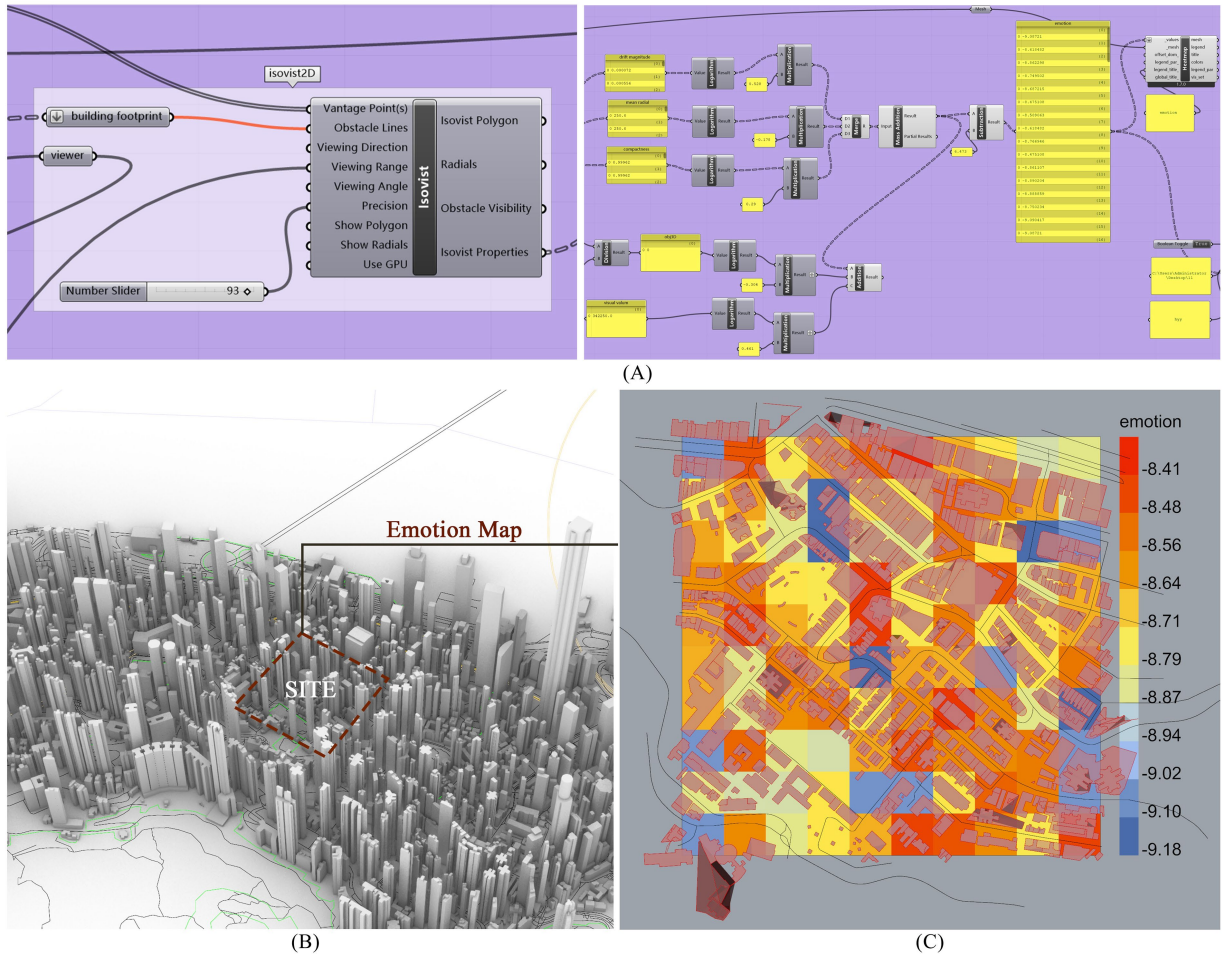
The process of generating emotional maps. **(A)** The generation processes of regional emotional maps; **(B)** Emotion map site selection area; **(C)** The presentation of emotion map.

**Figure 4:**
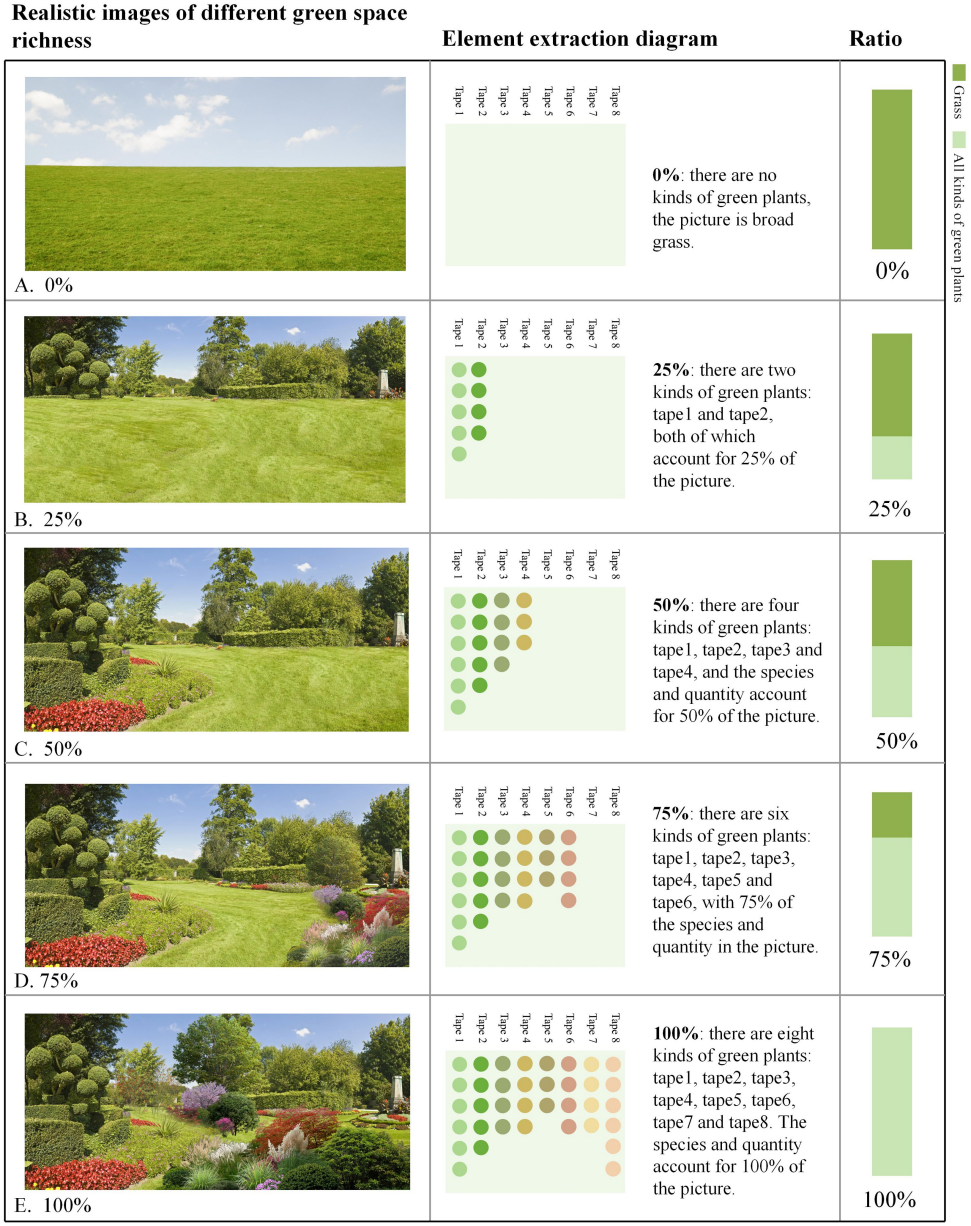
Green space richness at varying levels of vegetation density. **(A)** 0% green space richness, **(B)** 25% green space richness, **(C)** 75% green space richness; **(D)** 100% green space richness. **(E)** The types of elements included account for 100% of all element types present in the image.

**Figure 5:**
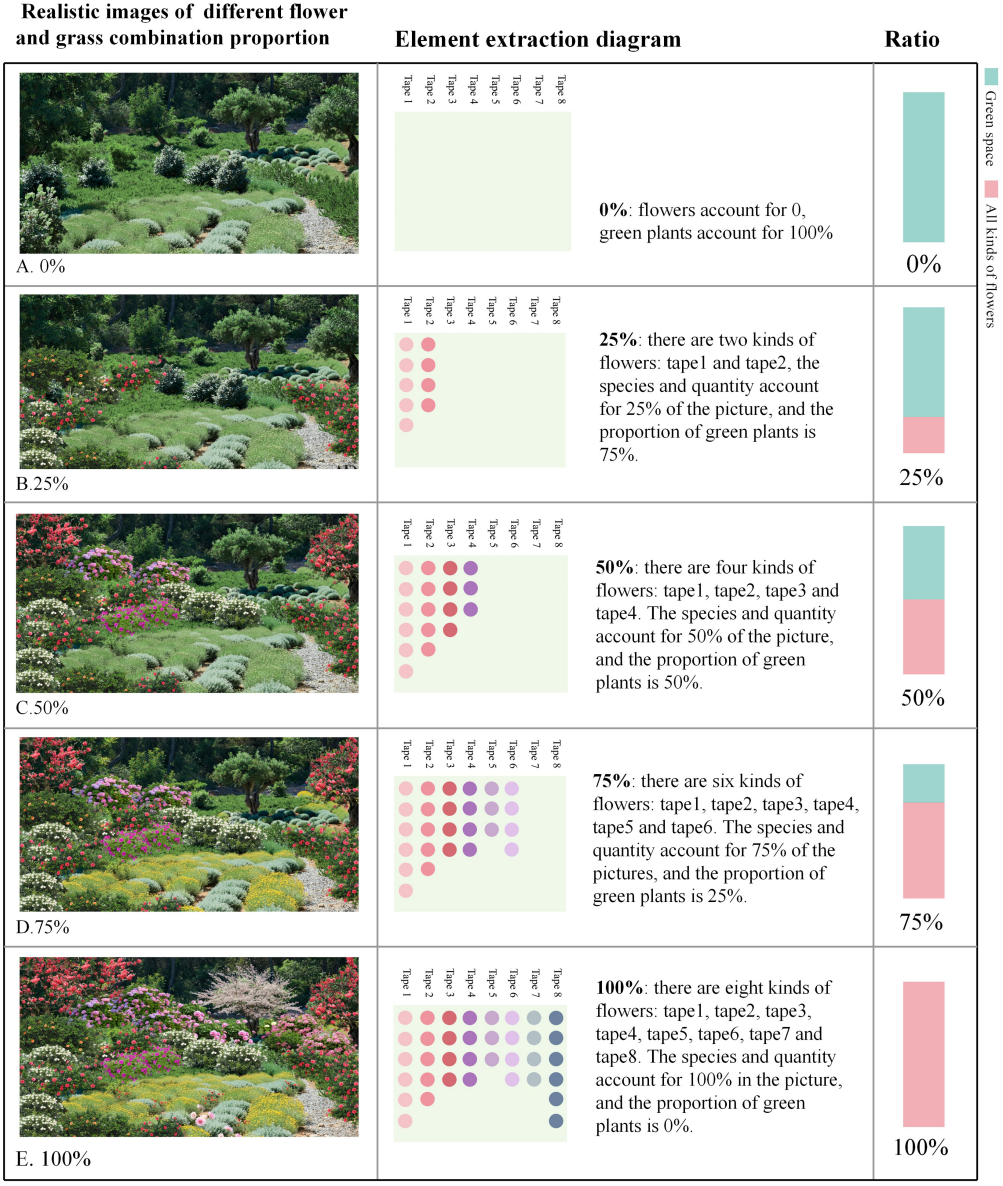
Proportions of flowers and plants in green spaces. **(A)** 0% ratio of flowers to grass, **(B)** 25% ratio of flowers to grass, **(C)** 75% ratio of flowers to grass; **(D)** 100% ratio of flowers to grass, **(E)** The elements included are all flowers, with no green plants.

**Figure 6:**
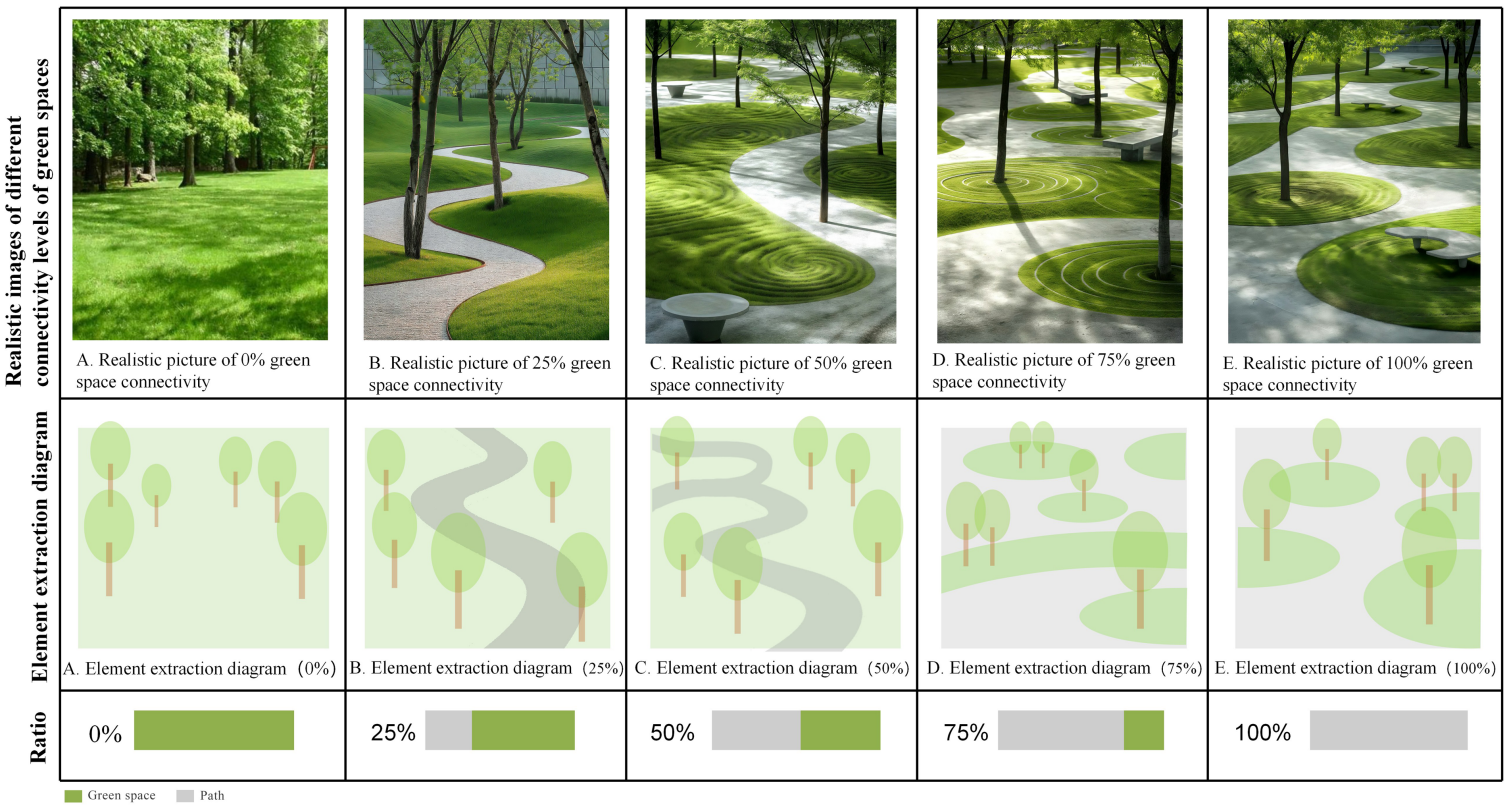
Green connectivity with different connectivity ratios. **(A)** 0% green connectivity, **(B)** 25% green connectivity, **(C)** green connectivity; **(D)** 100% green connectivity, **(E)** The paths in the green space are fully interconnected, with a connectivity rate of 100%.

In the emotional distribution map, low-emotion-value areas correspond to sections characterized by larger building exposure, higher line-of-sight obstruction rates, and lower levels of greenery distribution. To further investigate the preferences of residents in these low-emotion-value areas regarding community green spaces and their psychological responses, this study employed a questionnaire-based analysis (*N* = 455) (Figure 2). The questionnaire de-sign encompassed three key dimensions: demographic variable collection (age, gender, education level, frequency of green space use), landscape element preference evaluation (richness, plant combination, connectivity, spatial form), and emotion valence assessment. During the emotion testing phase, base maps of community green spaces were initially sourced from websites and subsequently edited using Photoshop software (Adobe 2018 version) to modify or add specific landscape elements. On the basis of this process, a total of 20 representative images were systematically selected and categorized into four groups, each comprising five images. Specifically, the first three groups corresponded to the following dimensions: green space richness, the flower-to-grass ratio, and connectivity, with the main element proportions set at 0, 25, 50, 75, and 100%. The fourth group focused on green space shape, featuring five representative forms: green spaces with height variations, curved green spaces, circular green spaces, rectangular green spaces, and zigzag-shaped green spaces. These images were employed for participants’ emotion and preference evaluations (Figures 3–6). The spaces presented in this study are based on real green spaces in Hong Kong and informed by on-site investigations. While the images have been stylized to isolate variables, key background elements have been retained to reflect Hong Kong’s urban structure and ensure correspondence with actual urban spaces. This method effectively balances internal validity (through variable control) with external validity (by reflecting local conditions).

(1) Preference test: All the images were divided into four groups, with each group containing five images. In the preference test, participants were required to select the image that evoked the strongest positive psychological response through intragroup comparisons. The test instructions were as follows: “Please carefully examine the scenes depicted in the images below and choose the one that feels most comfortable to you based on your personal preference.” Through this process, a single representative image was ultimately identified from each group to serve as the research outcome reflecting the group’s preference.
(2) Emotion test: Before the experiment, the participants needed to read and sign the informed consent form and personal information sheet. To ensure the scientific and accurate results of the test, this study provided a glossary containing definitions of key terms (exhilaration and relaxation) to minimize potential biases that may arise from differences in the understanding of these terms. During the experiment, the participants viewed five groups of pictures in sequence and considered their feelings when they were in the corresponding environment. They independently rated the degree of exhilaration and relaxation that each picture evoked on a 10-point scale (0–2 = dislike, 4–6 = neutral, 6–8 = slightly like, 8–10 = like). Each picture was presented to the subjects one by one in single-image display mode (each picture is displayed for at least 5 s) to maintain their concentration and ensure the consistency of the rating process. Additionally, participants were allowed to return and modify the ratings of the previous picture at any time to further enhance the accuracy and reliability of the assessment results (Figure 2).

The online questionnaire was hosted on the Questionnaire Star platform^1^ and was conducted between March 19, 2024, and November 19, 2024. The participants provided anonymous written consent, and a reward was offered for participation. The data were anonymized, and the authors did not have access to identifiable information about individual participants during or after data collection. Efforts were made to ensure the diversity and authenticity of the responses, with participants recruited via email, websites, social media, and online discussion forums. A total of 480 responses were received, of which 455 were considered valid, with an effective response rate of 94.79%.

### 2.3 Online questionnaire data analysis

This study utilized IBM SPSS Statistics 26.0 for Windows for data analysis. Based on immersive scene experiences, preference selection analysis was conducted on four dimensions of community green space forms (green space richness, flower and grass combination ratio, green space connectivity and shape), and by setting different element proportions ranging from 0 to 100%, the specific impacts of various green space features on exhilarating and relaxing moods were evaluated.

Data analysis methods include one-way analysis of variance (ANOVA), which is used to explore the influence of each dimension variable on emotions and to visualize the results to intuitively display the significant differences in means. Categorical variables are presented as frequencies and percentages. To further explore the relationships between personal preferences for community green space design combinations and demographic characteristics, three variables, namely, age, gender, and frequency of green space use, were selected to analyze their correlations with individual green space elements. The significance level for statistical analysis was set at *p* < 0.05 to determine whether there were statistically significant associations between different variables.

## 3 Results

### 3.1 Demographic characteristics of the sample

The demographic characteristics of the sample population exhibited a typical distribution pattern of urban residents (*N* = 455): the age range spanned from 18 to 65 years, with the core age group of 26 to 50 years constituting the majority at 62.4%. The educational level was predominantly a bachelor’s degree or above (57.8%), with junior high school education or below (33.6%), thereby ensuring sample heterogeneity. The gender ratio was relatively balanced (55.2% male and 44.8% female). The participants demonstrated a high-frequency usage pattern for green spaces, visiting, on average, 2 to 3 times per week. In terms of spatial utilization, there was a distinct clustering feature, which was primarily concentrated in squares (29.7%) and the remaining areas (24.3%) (Table 1).

**Table 1:** Demographic profile of the participants.

The proportion of various population groups		Educational background	Middle school	5.5%	High school	25.1%	College degree	11.6%	Bachelor’s degree	51.6%	Master’s degree	6.2%	Green space usage time	Early morning	25.7%	Early morning	16.4%	Afternoon	12.9%	Evening	23.3%	Late at night	21.7%	Green space usage frequency	None	2.2%	1 ~ 2 times	37.6%	3 ~ 4 times	42.2%	5 + times	18%	Age	18−	7%	18 ~ 25	11.9%	26 ~ 30	24.4%	31 ~ 40	6.2%	41 ~ 50	35.4%	51 ~ 60	12.3%	60+	2.9%	Gender	Male	55.2%	Female	44.8%	Types of activities on green spaces	Square	29.7%	Tussock	14.7%	Walkway	20.4%	Rest area	24.3%	Children’s entertainment area	10.8%

### 3.2 Preferences of the surveyed population

**Figure 7:**
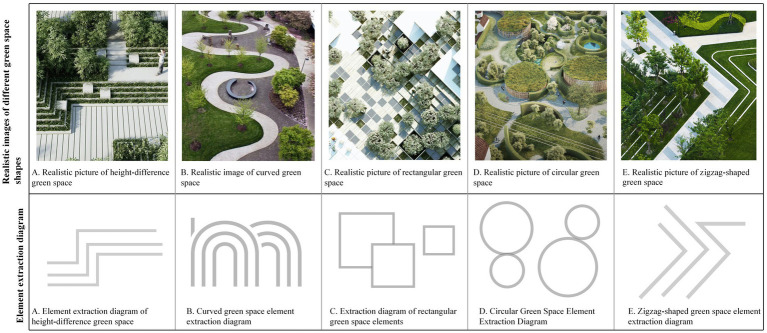
Different shapes of green spaces. **(A)** Height-difference green space, **(B)** curved green space, **(C)** rectangular green space; **(D)** Circular green space; **(E)** Zigzag-shaped green space.

The preference ratio chart of the crowd (Figure 7) indicates that, in terms of green space richness, the flower-to-grass combination ratio, and green space connectivity, people’s preferences exhibit consistent patterns: images with a 50% ratio are most favored, whereas those with a 0% ratio are least favored.

Specifically, with respect to green space richness, images with a 50% ratio received the highest preference, accounting for 30.5% of all selections, followed by those with a 75% ratio accounting for 25.7%. The images with a 0% ratio had a selection rate of only 7%, which is 23.5 percentage points lower than that of the most popular 50% images. In terms of the flower-to-grass combination ratio, images with a 50% ratio were most preferred, comprising 28.4% of all choices, which is 18.4 percentage points higher than the least popular 0% images. In terms of green space connectivity, images with a 50% ratio ranked first, with a selection rate of 29.5%, whereas those with a 0% ratio had a selection rate of only 9%, which is 20.5 percentage points lower than the highest value. Concerning green space shape preference, rectangular green spaces were the most favored form, accounting for 31% of all choices, followed by arc-shaped green spaces (23.3%), whereas broken-line shaped green spaces were the least favored, with a selection rate of 10.3%. These findings suggest that regular geometric shapes align more closely with the aesthetic preferences of the general public.

### 3.3 Emotional responses

#### 3.3.1 Comparative analysis of the average exhilaration scores

**Figure 8:**
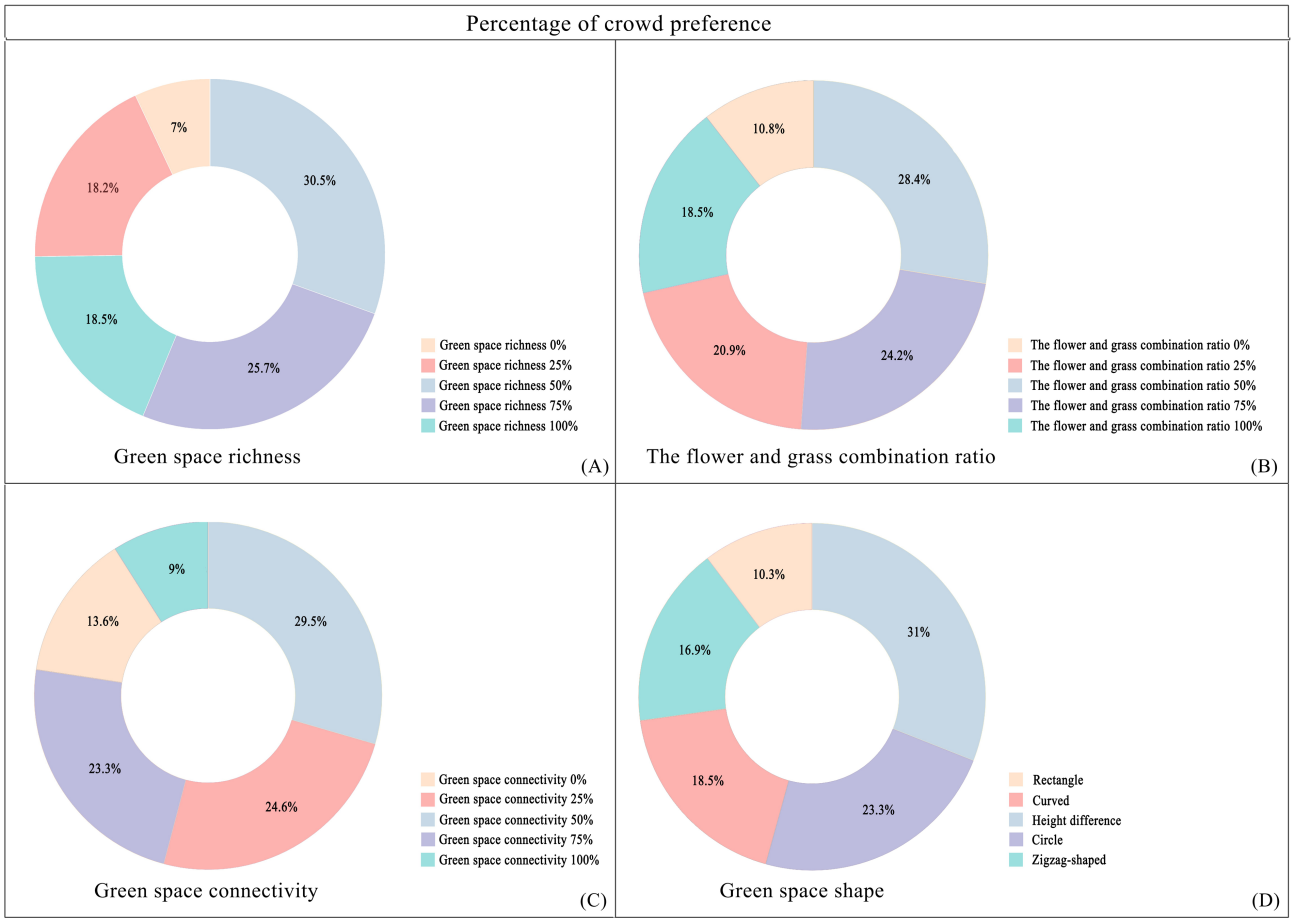
Population preference ratio chart. **(A)** The proportion of people’s preferences for different levels of green space diversity; **(B)** The proportion of people’s preferences for different combinations of flowers and plants; **(C)** The proportion of different population preferences for green space connectivity; **(D)** The proportion of people’s preferences for different shapes of green spaces.

There are significant variations in how different landscape elements influence improving people’s moods (Figure 8). (1) In terms of green space richness, 25% of the green space richness score was the highest (7.37 points), which was approximately 28% higher than the lowest value in the same dimension. The specific score order was 25% > 50% > 0% > 100% > 75%, indicating that a moderate green space density (25%) is most conducive to improving mood, whereas excessively high green space density (75 and 100%) may have negative effects. (2) In terms of the proportion of flower and grass combinations, the 50% ratio received the highest score (7.52 points), not only performing the best in this dimension but also achieving the highest score among all tested variables. The specific score order was 50% > 100% > 75% > 25% > 0%, indicating that a moderate combination of flower and grass plants (50%) was the most effective, whereas a complete lack of flowers (0%) was the least effective. (3) In terms of green space connectivity, a 0% green space connectivity score received the highest score (7.24 points), which was approximately 22.7% higher than the lowest score in the same dimension. The specific score order was 0% > 25% = 75% > 50% > 100%, indicating that low green space connectivity (0%) is more conducive to increasing the sense of pleasure, whereas high green space connectivity (100%) has a relatively weaker effect. (4) In terms of the shape of green spaces, arc-shaped spaces have the highest pleasure score (7.31 points), which is approximately 21.8% higher than the lowest value. The specific score order is as follows: curved green spaces > zigzag-shaped green spaces > green spaces with height differences > circular green spaces > rectangular green spaces. These results indicate that curved green spaces are more conducive to improving mood, whereas the effect of regular rectangular green spaces is relatively limited.

#### 3.3.2 Comparative analysis of the average relaxation scores

**Figure 9:**
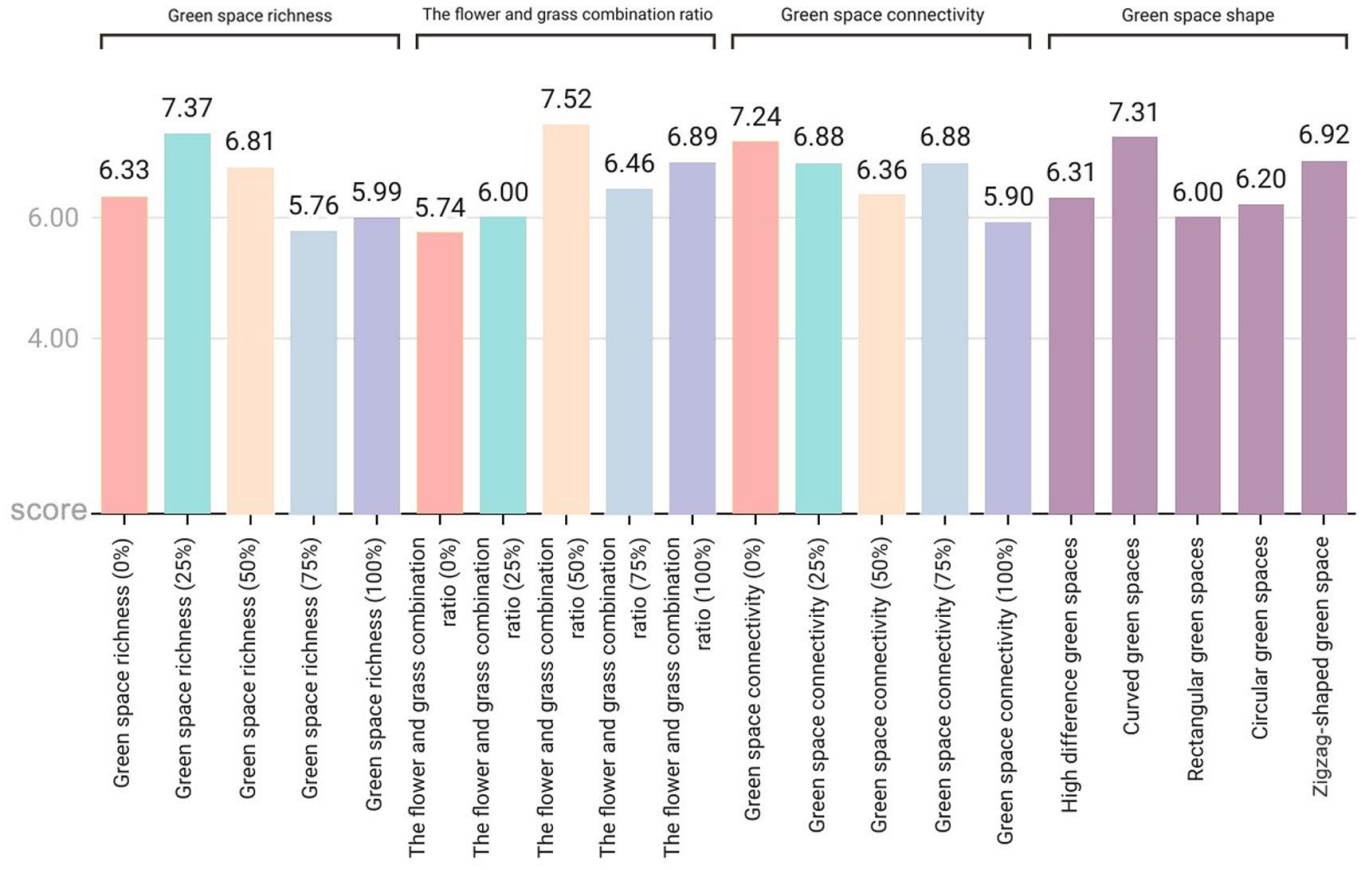
Average exhilaration score for each variable in the four dimensions of green space richness, flower and plant proportion, connectivity, and green space shape.

Research on the impact of green spaces on people’s relaxation levels (Figure 9) reveals the following: (1) In terms of green space richness, a richness level of 50% achieved the highest relaxation score (7.43 points), which was approximately 23.2% higher than the lowest value in the same dimension and represented the highest score among all tested variables. The specific ranking of scores was 50% > 100% > 25% > 75% > 0%. The environment with 0% green space richness had the poorest effect, indicating that a moderate level of green space richness (50%) was most conducive to relaxation. (2) Regarding the proportion of flowers and plants, the combination with 0% flowers achieved the highest relaxation score (7.07 points), which was approximately 19.6% higher than the lowest score. The specific ranking of the scores was 0% > 75% = 100% > 50% > 25%, suggesting that an environment with no flowers (0%) is most conducive to relaxation, whereas a combination of 25% flowers is less effective. (3) Concerning green space connectivity, a connectivity level of 25% achieved the highest relaxation score (7.07 points), which was approximately 14.6% higher than the lowest value. The specific score ranking was 25% > 75% > 100% > 0% > 50%, indicating that moderate green space connectivity (25%) is most conducive to relaxation, whereas 50% connectivity is the least ideal.

In terms of green space shape, rectangular green spaces achieved the highest relaxation score (7.28 points), which was approximately 14.8% higher than the lowest value. The specific score ranking was rectangular green spaces > zigzag-shaped green spaces > circular green spaces > green spaces with height differences > curved green spaces. Notably, these results contrast with the impact of the green space shape on the exhilaration, where curved green spaces performed the worst in terms of relaxation but the best in the exhilaration.

### 3.4 Influence of age on emotional responses

**Figure 10:**
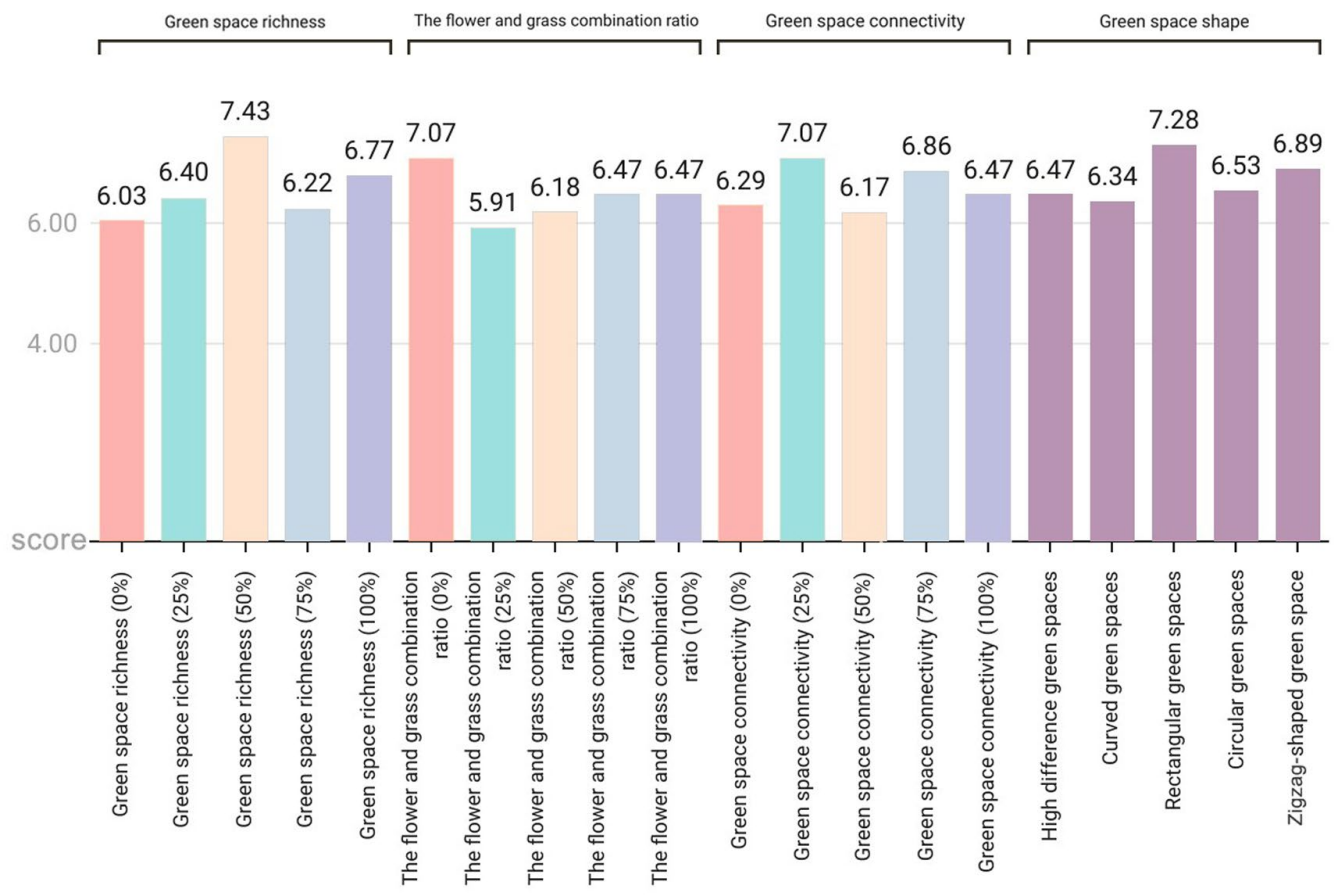
Average relaxation scores for each variable across the four dimensions: green space richness, flower and plant proportion, connectivity, and green space shape.

The analysis results revealed the general characteristics of the overall population but did not highlight the differences among different age groups. To further investigate the impact of age on landscape preferences, a grouped statistical analysis was performed on the emotional scores of the respondents across four dimensions—green space richness, the flower-to-grass ratio, green space connectivity, and green space shape (Figure 10). The findings indicated that age differences had a significant influence on individuals’ emotional responses to landscape elements, with each age group displaying distinct scoring patterns in these dimensions.

#### 3.4.1 Effects of different ages on exhilaration

In the analysis of mood-enhancing scores, the following characteristics were observed across different age groups (Figure 10):

(1) Green space richness: All age groups rated 25% green space richness as the highest score. Notably, the 51–60 age group assigned the lowest score (5.36 points) to 100% green space richness, whereas the other groups rated 75% green space richness as the lowest. This suggests that an overly dense green environment may significantly reduce the mood-enhancing effects for middle-aged and older adult individuals aged 51–60 years.
(2) Proportion of flowers and plants: All age groups agreed that a 50% combination of flowers and plants was most effective in improving mood. The absence of flowers (0% combination) received the lowest score across all groups, with the over 60 group scoring it at only 4.62 points. These findings indicate that a lack of flowers has a substantial negative effect on the emotional well-being of older adults.
(3) Green space connectivity: Except for teenagers under 18 years of age, all the other groups rated 0% green space connectivity as the highest score. In contrast, the teenage group rated 50% green space connectivity as the highest (7.25 points), suggesting that a community design promoting exploration and cross-road connectivity can effectively enhance teenagers’ moods. Additionally, all groups rated 100% green space connectivity as the lowest score, indicating that excessive connectivity may not be conducive to mood enhancement.
(4) Shape of green spaces: All age groups rated curved green spaces as the most favorable, indicating that curved shapes are most effective in enhancing emotional arousal. Among them, the over 60 group gave rectangular green spaces a notably low score of 4.62, below the median, reflecting a clear preference against overly regular geometric shapes. This result implies that these shapes may hinder the improvement of positive emotions among older adults.

#### 3.4.2 Effects of different ages on relaxation

In the analysis of relaxation scores, the following differences among age groups were observed (Figure 10):

(1) Green space richness: Young people aged 18–25 years gave the highest score (7.17) to 100% green space richness, indicating that a dense green environment enhances relaxation for this group. The other age groups preferred 50% green space richness, suggesting that moderate green density better supports relaxation for most individuals. The young age group had the lowest rating of 75% green space richness, whereas the other groups, particularly those over 60, had the lowest score (4.85) to 0% green space richness. These findings suggest that the absence of green space may significantly increase stress levels among older adults.
(2) Flower and grass proportion: Individuals aged 41–50 gave the highest score to a 75% flower and grass proportion, reflecting a preference for environments with higher floral content. In contrast, the other groups favored a 0% flower and grass proportion (pure grassland), indicating that expansive green landscapes better meet public relaxation needs. With respect to negative emotions, the older group over 60 had the lowest score for the 75% flower and grass proportion, whereas the other groups rated 25% as the least favorable. This implies that flower and grass proportions exceeding 25% may induce stress responses in certain groups, particularly among older adults, who are more sensitive to high floral proportions.
(3) Green space connectivity: Most groups preferred 25% green space connectivity, whereas adolescents gave the highest score (7.12) to 75% connectivity. This difference indicates that adolescents favor path designs with greater exploration opportunities during relaxation, whereas adults prefer moderately separated spatial layouts.
(4) Green space shape: Except for adolescents, all groups favored rectangular green spaces, highlighting the general applicability of regular shapes for relaxation. Adolescents, however, gave the highest score (6.98) to zigzag-shaped green spaces, which is consistent with their preference for exhilaration, suggesting a greater acceptance of nontraditional geometric shapes in this age group.

### 3.5 Factors affecting green space connectivity

The results of the variance analysis of the questionnaire data indicate that age, frequency of green space use, and gender significantly influence the invigoration emotion scores associated with green space connectivity (Table 2). Specifically, the invigoration emotion score for 0% green space connectivity was significantly negatively correlated with age (*p* < 0.05). As illustrated in Figure 7, the emotion score for this dimension decreases with increasing age (*p* = 0.039), suggesting that younger individuals prefer exploratory spaces with moderate path intersections (50% connectivity), whereas older adults perceive weaker emotional enhancement effects from moderately connected green spaces (50%).

**Table 2:** Analysis of variance (ANOVA) of the emotional impact of population age on green space connectivity (*p* < 0.05).

Green space connectivity	Age								
	Under 18 (*N* = 32)	18 ~ 25 (*N* = 54)	26 ~ 30 (*N* = 111)	31 ~ 40 (*N* = 161)	41 ~ 50 (*N* = 56)	51 ~ 60 (*N* = 28)	60 + (*N* = 13)	*F*	*P*
(0%)	6.75 ± 2.83	7.44 ± 2.13	7.5 ± 2.106	7.33 ± 2.43	7.07 ± 2.17	6.61 ± 2.69	6.46 ± 2.88	1.20	0.306
(25%)	6.56 ± 2.55	6.91 ± 2.48	7.14 ± 2.45	6.89 ± 2.61	6.95 ± 2.55	6.29 ± 2.62	6.23 ± 2.95	0.67	0.677
(50%)	7.25 ± 2.34	7.06 ± 2.48	6.41 ± 2.23	6.08 ± 2.49	6.27 ± 2.48	6.00 ± 2.75	5.46 ± 2.40	2.23	0.039**
(75%)	6.13 ± 2.92	7.17 ± 2.30	7.16 ± 2.49	6.93 ± 2.51	6.75 ± 2.57	6.39 ± 2.8	6.23 ± 2.86	1.15	0.331
(100%)	6.03 ± 2.02	5.93 ± 1.95	6.28 ± 2.26	5.74 ± 2.34	5.7 ± 2.231	5.89 ± 1.83	5.23 ± 2.92	0.96	0.451

**p <0.05.

Moreover, the invigoration emotion scores for 0, 50, and 100% green space connectivity are positively correlated with the frequency of green space use (*p* < 0.05) (Table 3). This finding underscores the individual differences in emotional regulation provided by green environments. With respect to gender differences, under the condition of 100% green space connectivity, there was a significant difference in invigoration emotion scores between genders (*p* < 0.05) (Table 4). Females scored significantly higher than males did (females: 6.13 ± 2.243; males: 5.72 ± 2.219), indicating that women are more likely to express positive emotions in highly connected green spaces, whereas men tend to men tend to show neither strongly positive nor strongly negative emotional responses (25) (as indicated by scores clustering around the midpoint of 5 on a 10-point scale). Further analysis of open-ended feedback indicates that urban green space activities hold greater importance for women compared to men (26). Male participants frequently associate highly connected green spaces with functional utility, such as efficient commuting routes (27), and show a preference for undeveloped green areas, like forests or regions located further from the city center (28). In contrast, women place greater emphasis on aesthetic and social dimensions, such as beautiful scenery and opportunities for social interaction, and demonstrate a stronger inclination toward well-maintained urban green spaces and recreational facilities (28).

**Table 3:** Analysis of variance (ANOVA) of the impact of the frequency of green space usage on the exhilaration score of green space connectivity (*p* < 0.05).

Green space connectivity	Frequency of green space usage					
	Almost not used (*N* = 10)	1 ~ 2 (*N* = 171)	3 ~ 4 (*N* = 192)	5 + (*N* = 82)	*F*	*P*
(0%)	6.9 ± 2.644	7.36 ± 2.251	7.44 ± 2.359	6.59 ± 2.424	2.828	0.038**
(25%)	6.4 ± 1.838	6.86 ± 2.332	7.15 ± 2.576	6.35 ± 2.916	1.996	0.114
(50%)	4.8 ± 2.781	6.58 ± 2.339	6.42 ± 2.431	5.95 ± 2.601	2.639	0.049**
(75%)	7.1 ± 2.685	6.8 ± 2.477	6.95 ± 2.601	6.85 ± 2.606	0.134	0.94
(100%)	4.7 ± 2.946	5.84 ± 2.187	6.19 ± 2.149	5.51 ± 2.369	2.96	0.032**

**p <0.05.

**Table 4:** Analysis of variance (ANOVA) of the impact of gender on the exhilaration score of green space connectivity (*p* < 0.05).

Green space connectivity	Gender			
	Male (*N* = 251)	Female (*N* = 204)	*T*	*P*
(0%)	7.26 ± 2.378	7.23 ± 2.323	0.151	0.88
(25%)	7.06 ± 2.497	6.65 ± 2.6	1.717	0.087
(50%)	6.42 ± 2.504	6.29 ± 2.384	0.559	0.577
(75%)	6.79 ± 2.532	6.99 ± 2.575	−0.821	0.412
(100%)	5.72 ± 2.219	6.13 ± 2.243	−1.975	0.049**

**p <0.05.

## 4 Discussion

This study investigates the influence of community green space landscapes on residents’ emotional health in the low-emotional-value urban areas of Hong Kong’s Central and Western District. Four key aspects—green space richness, the ratio of flower and grass combinations, connectivity, and shape—are analyzed to determine residents’ preferences for community green space landscapes and their associated emotional response mechanisms. These findings provide a scientific foundation for enhancing the quality of living environments.

### 4.1 Preferences and emotional responses

This study revealed that respondents’ preferences for green space richness, the proportion of flower and grass combinations, and connectivity presented significant commonalities: the pictures with a 50% element proportion were the most preferred, whereas those with a 0% element proportion were the least preferred (Figure 6). This finding aligns with Kaplan’s (29) attention restoration theory (ART), which posits that moderately complex visual patterns can elicit positive emotions, whereas minimalist or overly chaotic environments may weaken restorative effects. It is evident that people prefer community green spaces with evenly distributed landscape elements over those dominated by a single element. In terms of emotional enhancement, a 50% proportion of green space richness promoted higher levels of relaxation, and a 50% proportion of flower and grass combinations induced the highest level of emotional uplift (Figures 7, 8). Therefore, in the planning of community green spaces, it is essential to prioritize the balance of green space richness and the proportion of flower and grass combinations, with a 50% proportion being optimal for meeting people’s preferences for moderately complex visual patterns and enhancing emotional restoration effects.

This study revealed that overly dense and crowded trees may weaken people’s sense of uplifted mood (Figure 7). Research has also shown that the scarcity or overcrowding of green spaces can inhibit their positive emotional impact on residents (30). Moderate floral embellishment significantly enhances the overall exhilaration, which is consistent with prior findings that flowers elicit strong positive emotions (31), and grasslands with greater floral color diversity evoke stronger aesthetic preferences (4). Furthermore, increasing grassland area enhances relaxation, as green landscapes dominated by green plants with low floral coverage are more conducive to quiet reflection and optimal mood recovery (32). Simultaneously, the comfort and tranquility of green spaces provide a stress-relieving environment for individuals (33, 34). In environments where green space connectivity reaches 25%, people experience stronger relaxation (Figure 8), whereas areas with higher green space density and fewer road branches increased exhilaration (Figures 7, 8) (4). However, in Chinese cities, most lawns remain ornamental and rely heavily on forked roads to attract users. Future urban greening should reduce road forks, avoid excessive fragmentation, and enhance green space continuity, particularly in high-density urban areas. Additionally, cultivating tread-resistant grass species improves accessibility and utilization, enabling direct contact with nature (35–37). Research shows that curved green spaces significantly increase emotions in community environments, whereas rectangular green spaces promote relaxation. This finding holds practical significance for alleviating urban stress and promoting mental health through customized green space design. Future community green space planning could integrate the advantages of both curved and rectangular designs to increase positive emotions, create serene environments, and contribute to achieving the United Nations’ Sustainable Development Goals related to good health and well-being as well as sustainable cities and communities.

### 4.2 Influence of age on emotional responses

Previous studies have demonstrated that the association between green environments and health benefits is particularly pronounced among specific demographic groups, such as minors and older adult individuals (38, 39). This study reveals distinct differences in emotional responsiveness to green space features across different age groups:

(1) Minors: Dense green environments are more effective at promoting relaxation among minors (Figure 9), whereas community green spaces with increased crossroads and greater exploratory potential increase their exhilaration. Research indicates a positive correlation between green environments and positive emotions in adolescents (40). These environments not only provide attractive social spaces (41) but also fulfill minors’ needs for exploring new experiences (42). For this group, designing curved road networks, open lawns, and interactive landscape installations (e.g., plant mazes) can stimulate exploration interest and foster social interaction. In terms of vegetation configuration, a balanced multilevel structure (trees, shrubs, flowers, and grass) should be maintained, with open grasslands retained to meet relaxation needs and visual richness enhanced through strategic floral embellishments.
(2) Older adult: Compared with grass, environments with a slightly greater proportion of flowers significantly improve relaxation among older adult individuals (Figure 9). Conversely, overly dense green spaces, flower scarcity, and overly regular rectangular layouts notably reduce their exhilaration. This finding may stem from changes in emotional expression intensity among older adult individuals, who tend to amplify positive or negative emotions (43, 44). Studies have shown that the presence of flowers positively influences emotions (31), and the interaction between flowers and plants reduces perceived stress and enhances physical and mental well-being in older adults (45). With the growing trend of population aging, current community green space designs have yet to fully adapt to the needs of an aging population (46, 47). To address this gap, age-friendly green space design principles could be integrated into urban renewal standards, incorporating diverse functional areas such as children’s exploration zones and older adult rehabilitation gardens. For older adult individuals, increasing the proportion of flowers in green spaces (30–40%), prioritizing natural curvilinear layouts over rigid rectangles, introducing fragrant plants (e.g., lavender) and soft vegetation (e.g., moss), and combining barrier-free pathways with seating arrangements to enhance multisensory rehabilitation experiences are recommended.

### 4.3 Other influencing factors

Gender differences significantly influence emotional orientation toward the connectivity of community green spaces (Table 4). Research shows that women are more likely to express positive emotions in green environments, whereas men typically exhibit a more neutral response (Table 4) (25). Evolutionary psychology further posits that women, due to their historical roles in gathering and nurturing infants, may exhibit heightened sensitivity to natural elements (48), which may be attributed to evolutionary theory suggesting that women possess a stronger ability to perceive and remember plant complexity (49), potentially leading to greater increase in happiness from green spaces (50). This gender disparity not only highlights the diversity in how individuals interact with natural environments but also offers new insights into studying emotional responses in green space usage. These findings resonate with Sustainable Development Goal 5 (Gender Equality), underscoring the importance of gender-inclusive green space design that accommodates diverse preferences. For example, integrating functional facilities, such as exercise trails, with aesthetically enriched landscapes can reconcile gender-specific needs and foster equitable access to mental health benefits (51).

Therefore, when planning future community green spaces, gender differences should be carefully considered. Given women’s heightened sensitivity to natural environments, multilevel vegetation structures (e.g., combinations of trees, shrubs, and flowers) can be incorporated to increase visual complexity and encourage exploration. Additionally, spaces for social interaction (e.g., shared gardens and rest pavilions) can be established to foster community cohesion and emotional exchange. For men, introducing functional facilities (e.g., sports trails and fitness equipment) can improve the practicality of green spaces, meet their preferences for neutral environments and enhance the overall user experience.

### 4.4 Policy implications

The results of this study are highly significant for advancing the United Nations Sustainable Development Goals (SDGs), particularly within the context of urban planning priorities in Hong Kong. The Hong Kong Special Administrative Region (SAR) government has explicitly highlighted SDG 3 (Good Health and Well-being) and SDG 11 (Sustainable Cities and Communities) in its strategic plan, “Hong Kong 2030+: Planning Vision and Strategy Beyond 2030.” This plan aims to achieve these goals by enhancing the livability, public health, and environmental sustainability of high-density urban areas. The research findings indicate that green space standards informed by population preferences should be integrated into urban planning policies. For example, the optimal values of 50% vegetation coverage and 25% connectivity identified in this study can serve as critical references for municipal residential green space design guidelines. Local governments could establish these thresholds through legislation to ensure that communities provide balanced environments conducive to emotional recovery and mental well-being.

Moreover, the findings on gender and age differences further underscore the importance of inclusive design principles, closely resonating with Sustainable Development Goals 5 (gender equality) and 10 (reduced inequalities). To translate these insights into actionable policies, future efforts could focus on strengthening collaboration with municipal authorities and embedding evidence-based green space standards (such as optimal vegetation ratios and connectivity thresholds) into Hong Kong’s overall greening master plan. These initiatives not only contribute to localizing the SDGs but also position Hong Kong as a global exemplar for sustainable high-density urban development.

### 4.5 Limitations

Although this study focuses on four key dimensions of green space design, there may still be additional emotional influence factors that have not been considered. Future research will delve deeper into people’s preferences for community green space landscapes and their emotional responses from multiple angles, aiming to design green spaces with greater comprehensive value and practical significance, thereby providing a more comprehensive understanding of the mechanisms by which green spaces impact emotions. In addition, future research endeavors will focus on gathering a more extensive and representative dataset, which will substantially enhance the generalizability and robustness of the research findings.

## 5 Conclusion

**Figure 11:**
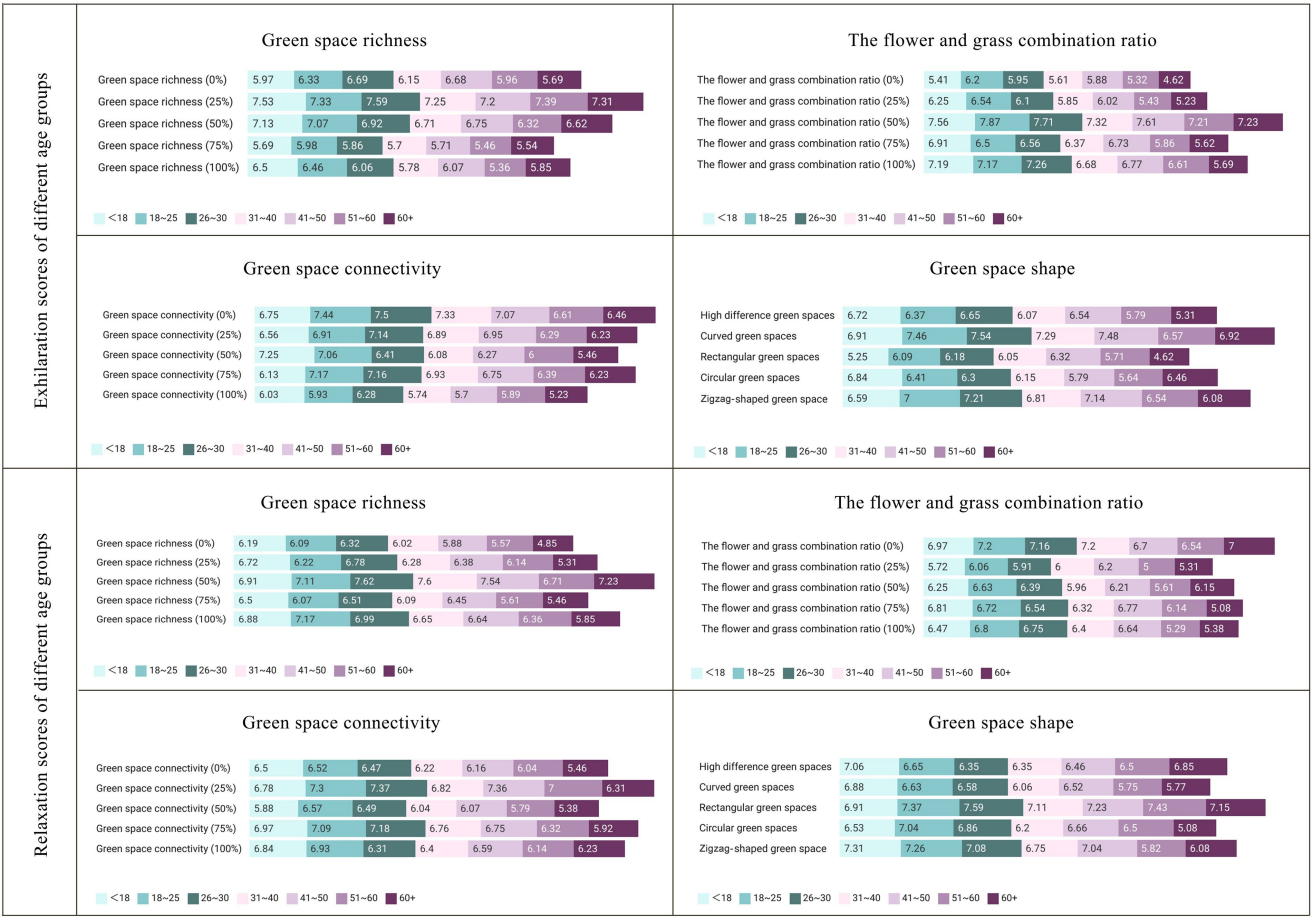
Exhilaration and relaxation scores for various dimensional variables across the different age groups.

This study employs isovist analysis to reveal the spatial distribution characteristics of urban residents’ emotions in the Central and Western District of Hong Kong, and systematically investigates the influence of community green space landscape design in low-emotional-value areas on residents’ emotional health, revealed the effects of four key dimensions—green space richness, the proportion of flowers and plants, connectivity, and shape—on emotional regulation. The results showed that the design of community green spaces can significantly affect the emotional state of residents and that their mental health can be improved through a reasonable landscape layout (Figure 11).

**Figure 12:**
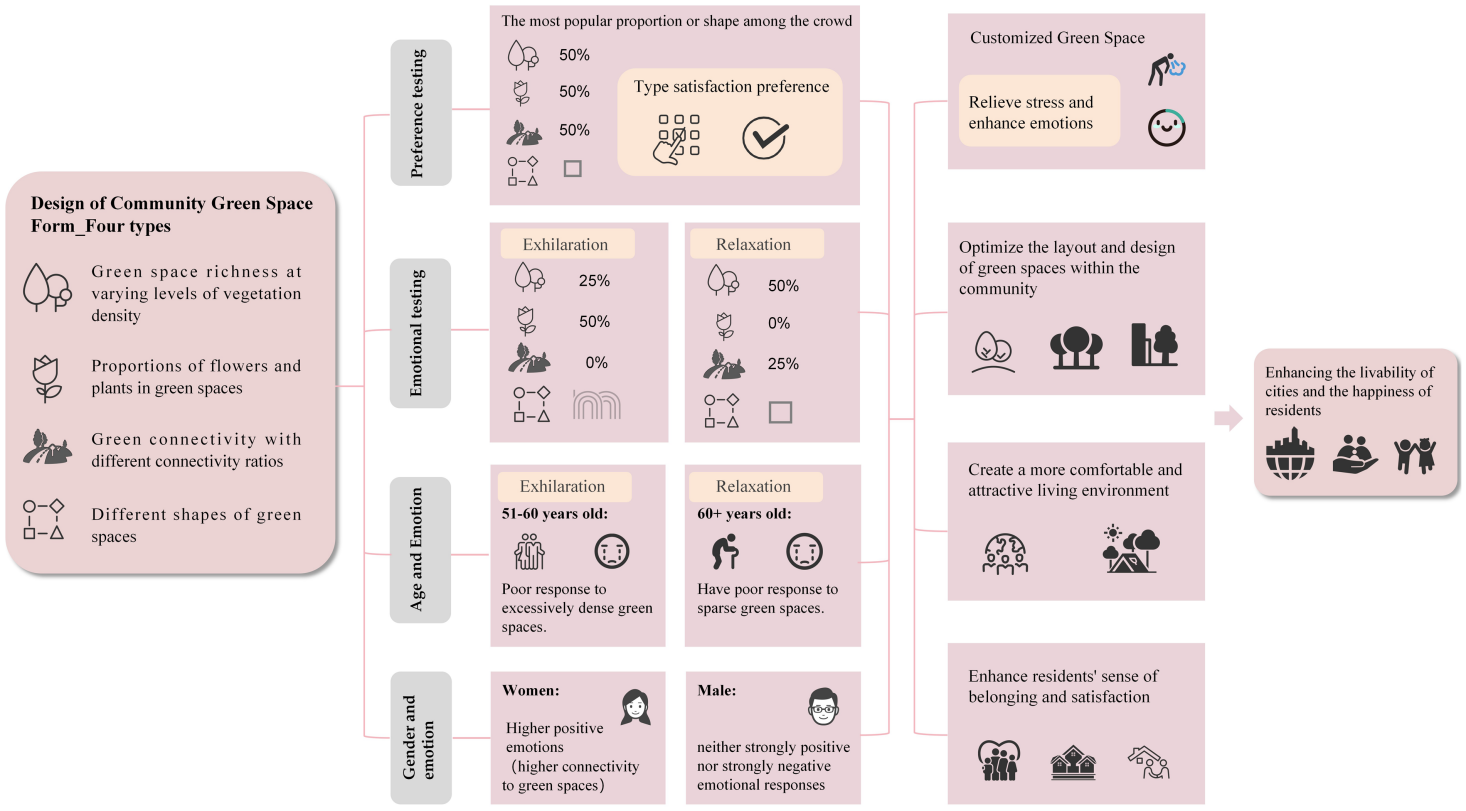
Conclusion summary framework.

Research has shown that an appropriate increase in the proportion of flowers can enhance people’s positive emotions, whereas an increase in the proportion of green spaces can enhance people’s relaxation levels. A 50% green space richness and a 50% proportion of flowers and plants are the most popular and can effectively enhance residents’ relaxation and exhilaration, in line with the theory of moderate-complexity visual patterns. Curved green spaces help to enhance mood, whereas rectangular green spaces are more conducive to generating relaxed emotions. Therefore, community green space planning should prioritize a 50% design ratio and combine the advantages of curved and rectangular green spaces to optimize emotional recovery effects. In addition, moderate green space connectivity (25%) can significantly enhance relaxation levels, whereas high connectivity (100%) is not conducive to the generation of positive emotions, suggesting that future UGS construction should reduce road forks and enhance the coherence of green spaces (Figure 12).

There are significant differences in emotional responses to green spaces across different age groups. Younger participants show a stronger preference for dense greenery and exploratory path designs, whereas older adults favor environments with a greater proportion of flowers relative to grass and tend to exhibit negative emotions when exposed to overly dense or rigidly rectangular green spaces. Therefore, the design of community green spaces should fully account for the distinct needs of various age groups. For example, interactive landscape installations can be incorporated for younger individuals, whereas green spaces featuring a moderately increased floral proportion and natural curvilinear layouts can be provided for older adults. Moreover, gender differences play a critical role in shaping emotional responses. Women are more likely to express positive emotions in green environments, whereas men typically display neither strongly positive nor strongly negative emotional responses. Future planning and design should comprehensively integrate multilevel vegetation configurations with social spaces to address women’s heightened sensitivity to natural environments. Simultaneously, functional facilities such as exercise trails and fitness equipment can be introduced to enhance the usability and appeal of green spaces for men.

In conclusion, this study provides a scientific basis for constructing community green spaces with therapeutic functions, assisting urban planners and community managers in better addressing the needs of different age and gender groups while enhancing residents’ sense of happiness and belonging. Furthermore, this research introduces a novel intervention approach for mental health professionals by encouraging patients to engage with and enjoy green space environments as a means to regulate emotions and alleviate stress. This study establishes a robust theoretical and practical foundation for customized community green space design and sustainable urban development. Future research should aim to expand the sample size, explore additional design elements, and examine cross-cultural variations in greater depth, thereby advancing the understanding of the relationship between green spaces and emotions and offering more comprehensive guidance for global urban green space planning.

## Data Availability

The original contributions presented in the study are included in the article/Supplementary material, further inquiries can be directed to the corresponding author.

## Footnotes

^1^ https://www.wjx.cn

## References

[1] Marsland AL Cohen S Rabin BS Manuck SB. Trait positive affect and antibody response to hepatitis B vaccination. Brain Behav Immun. (2006) 20:261–9. doi: 10.1016/j.bbi.2005.08.009, PMID: 16293394

[2] Higgs M Dulewicz V. Antecedents of well-being: a study to examine the extent to which personality and emotional intelligence contribute to well-being. Int J Hum Resour Manage. (2014) 25:718–35. doi: 10.1080/09585192.2013.815253

[3] Lee BX Kjaerulf F Turner S Cohen L Donnelly PD Muggah R. Transforming our world: implementing the 2030 agenda through sustainable development goal indicators. J Public Health Policy. (2016) 37:13–31. doi: 10.1057/s41271-016-0002-7 27638240

[4] Hao J Gao T Qiu L. How do species richness and colour diversity of plants affect public perception, preference and sense of restoration in urban green spaces? Urban For Urban Green. (2024) 100:128487. doi: 10.1016/j.ufug.2024.128487

[5] Ulrich R. S. View through a window may influence recovery from surgery. Science. (1984) 224:420–421. 6143402 10.1126/science.6143402

[6] Wang P Wang M Shan J Liu X Jing Y Zhu H. Association between residential greenness and depression symptoms in Chinese community-dwelling older adults. Environ Res. (2024) 243:117869. doi: 10.1016/j.envres.2023.117869, PMID: 38070849

[7] Allahyar M Kazemi F. Effect of landscape design elements on promoting neuropsychological health of children. Urban Forestry Urban Greening. (2021) 65:127333. doi: 10.1016/j.ufug.2021.127333

[8] Wang Y Zhang Q Li J. Effect of plantscape preference on the psychological recovery of university students: based on the mediating effect of prototype landscape consciousness. Urban For Urban Green. (2023) 88:128088. doi: 10.1016/j.ufug.2023.128088

[9] Robinson N. The planting design handbook. 3rd ed. Routledge: Taylor & Francis Group. (2016).

[10] Tamosiunas A Grazuleviciene R Luksiene D Dedele A Reklaitiene R Baceviciene M. Accessibility and use of urban green spaces, and cardiovascular health: findings from a Kaunas cohort study. Environ Health. (2014) 13:1–11. doi: 10.1186/1476-069X-13-20 24645935 PMC4000006

[11] Mitchell RJ Richardson EA Shortt NK Pearce JR. Neighborhood environments and socioeconomic inequalities in mental well-being. Am J Prev Med. (2015) 49:80–4. doi: 10.1016/j.amepre.2015.01.017, PMID: 25911270

[12] Wang H Tassinary LG Newman GD. Developing the health effect assessment of landscape (HEAL) tool: assessing the health effects of community greenspace morphology design on non-communicable diseases. Landsc Urban Plann. (2024) 244:104990. doi: 10.1016/j.landurbplan.2023.104990 PMC12705061 41404354

[13] Hung SH Chang CY. Designing for harmony in urban green space: linking the concepts of biophilic design, environmental qi, restorative environment, and landscape preference. J Environ Psychol. (2024) 96:102294. doi: 10.1016/j.jenvp.2024.102294

[14] Alcock I White MP Wheeler BW Fleming LE Depledge MH. Longitudinal effects on mental health of moving to greener and less green urban areas. Environ Sci Technol. (2014) 48:1247–55. doi: 10.1021/es403688w, PMID: 24320055

[15] Van den Berg AE Jorgensen A Wilson ER. Evaluating restoration in urban green spaces: does setting type make a difference? Landsc Urban Plann. (2014) 127:173–81. doi: 10.1016/j.landurbplan.2014.04.012

[16] Triguero-Mas M Dadvand P Cirach M Martínez D Medina A Mompart A. Natural outdoor environments and mental and physical health: relationships and mechanisms. Environ Int. (2015) 77:35–41. doi: 10.1016/j.envint.2015.01.012, PMID: 25638643

[17] Gola M Botta M Lisa D’Aniello A Capolongo S. How breaks in nature can affect the users’ wellbeing: an experience-based survey during the lockdown (COVID-19): strategies for healthy and resilient green areas in our cities. In: Cheshmehzangi A Sedrez M Zhao H Li T Heath T Dawodu A, editors. Resilience vs pandemics. Urban sustainability. Singapore: Springer (2024)

[18] Sharafatmandrad M Mashizi AK. Visual value of rangeland landscapes: a study based on structural equation modeling. Ecol Eng. (2020) 146:105742. doi: 10.1016/j.ecoleng.2020.105742

[19] Cai K Huang W Lin G. Bridging landscape preference and landscape design: a study on the preference and optimal combination of landscape elements based on conjoint analysis. Urban Forestry Urban Green. (2022) 73:127615. doi: 10.1016/j.ufug.2022.127615

[20] Van den Berg AE Koole SL Van Der Wulp NY. Environmental preference and restoration:(how) are they related? J Environ Psychol. (2003) 23:135–46. doi: 10.1016/S0272-4944(02)00111-1

[21] Veitch J Salmon J Deforche B Ghekiere A Van Cauwenberg J Bangay S. Park attributes that encourage park visitation among adolescents: a conjoint analysis. Landsc Urban Plann. (2017) 161:52–8. doi: 10.1016/j.landurbplan.2016.12.004

[22] Richardson EA Pearce J Mitchell R Kingham S. Role of physical activity in the relationship between urban green space and health. Public Health. (2013) 127:318–24. doi: 10.1016/j.puhe.2013.01.004, PMID: 23587672

[23] Nordh H Hartig T Hagerhall CM Fry G. Components of small urban parks that predict the possibility for restoration. Urban For Urban Green. (2009) 8:225–35. doi: 10.1016/j.ufug.2009.06.003

[24] Xiang L Cai M Ren C Ng E. Modeling pedestrian emotion in high-density cities using visual exposure and machine learning: tracking real-time physiology and psychology in Hong Kong. Build Environ. (2021) 205:108273. doi: 10.1016/j.buildenv.2021.108273

[25] Wei D Liu M Grekousis G Wang Y Lu Y. User-generated content affects urban park use: analysis of direct and moderating effects. Urban For Urban Green. (2023) 90:128158. doi: 10.1016/j.ufug.2023.128158

[26] Schipperijn J Ekholm O Stigsdotter UK Toftager M Bentsen P Kamper-Jørgensen F. Factors influencing the use of green space: results from a Danish national representative survey. Landsc Urban Plann. (2010) 95:130–7. doi: 10.1016/j.landurbplan.2009.12.010

[27] Ruan SN Zhang K. On public recreational preference of greenway based on stated-preference method——a case study of Chengdu. J Southwest China Norm Univ. (2021) 46:99–105. doi: 10.13718/j.cnki.xsxb.2021.01.016

[28] Bąkowska-Waldmann E Piniarski W. Gender-specific preferences regarding urban green areas. Quaest Geogr. (2023) 42:23–41. doi: 10.14746/quageo-2023-0037

[29] Kaplan S. The restorative benefits of nature: toward an integrative framework. J Environ Psychol. (1995) 15:169–82. doi: 10.1016/0272-4944(95)90001-2

[30] Wang R Browning MH Qin X He J Wu W Yao Y. Visible green space predicts emotion: evidence from social media and street view data. Appl Geogr. (2022) 148:102803. doi: 10.1016/j.apgeog.2022.102803

[31] Haviland-Jones J Rosario HH Wilson P McGuire TR. An environmental approach to positive emotion: flowers. Evol Psychol. (2005) 3:147470490500300109. doi: 10.1177/147470490500300109

[32] Hoyle H Hitchmough J Jorgensen A. All about the “wow factor”? The relationships between aesthetics, restorative effect and perceived biodiversity in designed urban planting. Landsc Urban Plann. (2017) 164:109–23. doi: 10.1016/j.landurbplan.2017.03.011

[33] Liu S Su C Yang R Zhao J Liu K Ham K. Using crowdsourced big data to unravel urban green space utilization during COVID-19 in Guangzhou, China. Land. (2022) 11:990. doi: 10.3390/land11070990

[34] Wan C Shen GQ Choi S. Eliciting users’ preferences and values in urban parks: evidence from analyzing social media data from Hong Kong. Urban For Urban Green. (2021) 62:127172. doi: 10.1016/j.ufug.2021.127172

[35] Yang F Ignatieva M Larsson A Xiu N Zhang S. Historical development and practices of lawns in China. Environ Hist. (2019) 25:23–54. doi: 10.3197/096734018X15137949592098

[36] Yang FP Ignatieva M Larsson A Zhang SX Ni N. Public perceptions and preferences regarding lawns and their alternatives in China: a case study of Xi’an. Urban For Urban Green. (2019) 46:126478. doi: 10.1016/j.ufug.2019.126478

[37] Yang F Ignatieva M Wissman J Ahrné K Zhang S Zhu S. Relationships between multi-scale factors, plant and pollinator diversity, and composition of park lawns and other herbaceous vegetation in a fast growing megacity of China. Landsc Urban Plann. (2019) 185:117–26. doi: 10.1016/j.landurbplan.2019.02.003

[38] Taylor AF Kuo FE Sullivan WC. Views of nature and self-discipline: evidence from inner city children. J Environ Psychol. (2002) 22:49–63. doi: 10.1006/jevp.2001.0241

[39] Maas J Verheij RA Groenewegen PP De Vries S Spreeuwenberg P. Green space, urbanity and health: how strong is the relation? J Epidemiol Community Health. (2006) 60:587–92. doi: 10.1136/jech.2005.043125, PMID: 16790830 PMC2566234

[40] Xiang Z Luo X Zheng R Jiang Q Zhu K Feng Y. Associations of greenness surrounding schools and self-reported depressive and anxiety symptoms in Chinese adolescents. J Affect Disord. (2022) 318:62–9. doi: 10.1016/j.jad.2022.08.095, PMID: 36058356

[41] Putra IGNE Astell-Burt T Cliff DP Vella SA Feng X. Do physical activity, social interaction, and mental health mediate the association between green space quality and child prosocial behaviour? Urban For Urban Green. (2021) 64:127264. doi: 10.1016/j.ufug.2021.127264

[42] Taczanowska K Tansil D Wilfer J Jiricka-Pürrer A. The impact of age on people's use and perception of urban green spaces and their effect on personal health and wellbeing during the COVID-19 pandemic—a case study of the metropolitan area of Vienna, Austria. Cities. (2024) 147:104798. doi: 10.1016/j.cities.2024.104798

[43] Liu P Liu M Xia T Wang Y Wei H. Can urban forest settings evoke positive emotion? Evidence on facial expressions and detection of driving factors. Sustainability. (2021) 13:8687. doi: 10.3390/su13168687

[44] Zhang J Yang Z Chen Z Guo M Guo P. Optimizing urban forest landscape for better perceptions of positive emotions. Forests. (2021) 12:1691. doi: 10.3390/f12121691

[45] Chan HS Chu HY Chen MF. Effect of horticultural activities on quality of life, perceived stress, and working memory of community-dwelling older adults. Geriatr Nurs. (2022) 48:303–14. doi: 10.1016/j.gerinurse.2022.10.016, PMID: 36347114

[46] Subramanian D Jana A. Assessing urban recreational open spaces for the elderly: a case of three Indian cities. Urban For Urban Green. (2018) 35:115–28. doi: 10.1016/j.ufug.2018.08.015

[47] Li ZZ Zhen F Xu HX. Spatial matching evaluation of supply and demand of elderly service facilities based on multi-source data – taking Nanjing as an example. Mod City Res. (2022) 8:8–15.

[48] Hoyle H Jorgensen A Hitchmough JD. What determines how we see nature? Perceptions of naturalness in designed urban green spaces. People Nat. (2019) 1:167–80. doi: 10.1002/pan3.19

[49] Silverman I. Eals M. (1992). Sex differences in spatial abilities: evolutionary theory and data. In Portions of this paper were presented at the meetings of the International Society for Human Ethology in Binghamton, NY, Jun 1990, the human behavior and evolution Society in Los Angeles, CA, Aug 1990, and the European sociobiological Society in Prague, Czechoslovakia, Aug 1991. Oxford, United Kingdom: Oxford University Press.

[50] Feng X Xiong J Tang Z. To be rational or sensitive? The gender difference in how textual environment cue and personal characteristics influence the sentiment expression on social media. Telemat Inform. (2023) 80:101971. doi: 10.1016/j.tele.2023.101971

[51] Sillman D Rigolon A Browning MH McAnirlin O. Do sex and gender modify the association between green space and physical health? A systematic review. Environ Res. (2022) 209:112869. doi: 10.1016/j.envres.2022.112869, PMID: 35123971

