# Supplementary material for: From emotional map to design criteria: verification of the correlation between community green space form and emotional health of high-density urban residents
Source: Front Public Health. 2025 Jul 15;13:1617294. doi: 10.3389/fpubh.2025.1617294 (PMC12306530; doi:10.3389/fpubh.2025.1617294)
Supplement: Supplementary file 1 [file Data_Sheet_1.zip › Survey questionnaire content and raw data/Survey Questionnaire on Community Green Space Design Preferences.docx]

**Survey Questionnaire on Community Green Space Design Preferences**

Dear interviewee:

Hello! We are conducting a study on the emotional impact of community green space design on people, aiming to explore how green space environments promote residents' mental health and happiness. Your answer will provide valuable reference for our research. This questionnaire does not involve personal privacy, and the information collected is only for academic research purposes. Thank you for your participation and support!

1. Your age: [Single choice question] *

| ○＜18 | ○18~25 | ○26~30 | ○31~40 |
| --- | --- | --- | --- |
| ○41~50 | ○51~60 | ○60+ |  |

2. Your gender: [Single choice question] *

| ○Man | ○Woman |
| --- | --- |
|  |  |

3. Your education background: [Single choice question] *

| ○Junior high school and below |
| --- |
| ○High school/vocational school |
| ○college degree |
| ○bachelor's degree program |
| ○Graduate students and above |

4. What is the average frequency of using community green spaces per week? [Single choice question]*

| ○Almost not used |
| --- |
| ○1-2 |
| ○3-4 |
| ○5+ |

5. When do you usually use community green spaces? [Multiple Choice Question] *

| □Morning（5:00~9:00） |
| --- |
| □Morning（9:00~12:00） |
| □Afternoon |
| □Evening |
| □At night |

6. What type of community green space do you usually engage in activities on? [Multiple Choice Question] *

| □Square |
| --- |
| □Grass |
| □Pedestrian or running track |
| □Rest area |
| □Children's entertainment area |

**Part 1:** Please choose your favorite pair of the following pictures according to your preferences, based on the quality of your inner feelings when immersed in the scene of the following pictures.

7. When you are immersed in the scene of the following picture, please choose the scene with the best feeling according to your own preference [Single choice question] *

| 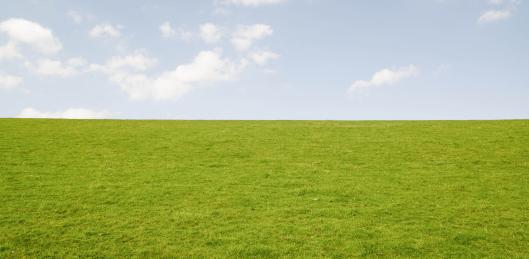 ○A. | 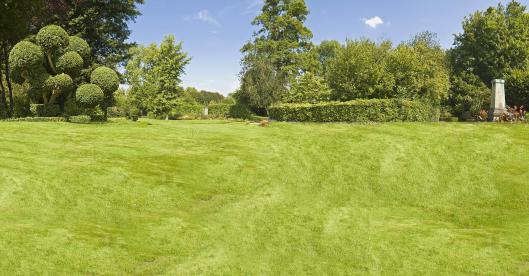 ○B. |
| --- | --- |
| 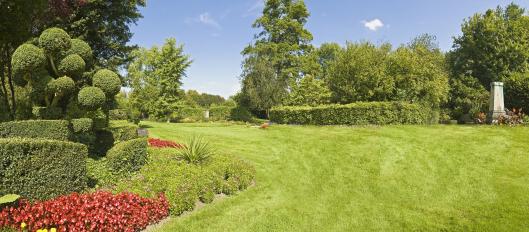 ○C. | 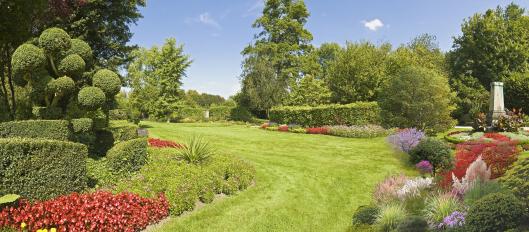 ○D. |
| 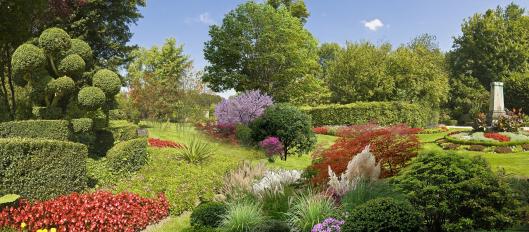 ○E. | |

8. When you are immersed in the scene of the following picture, please choose the scene with the best feeling according to your own preference [Single choice question] *

| 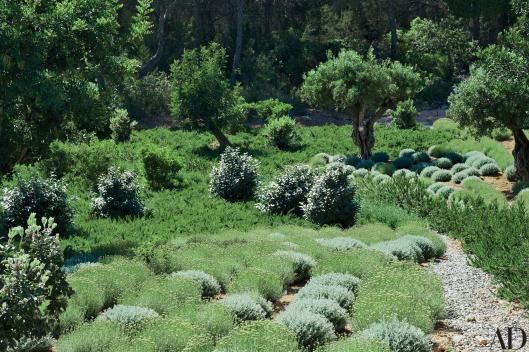 ○A. | 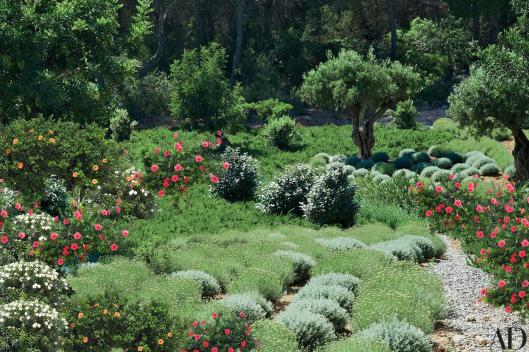 ○B. |
| --- | --- |
| 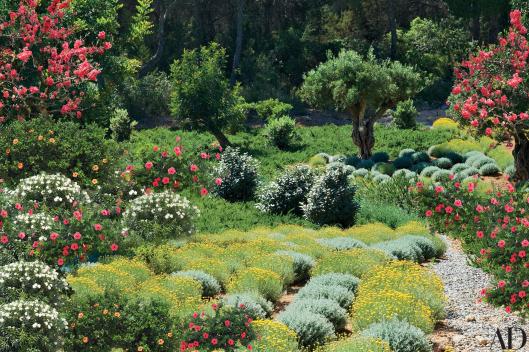 ○C. | 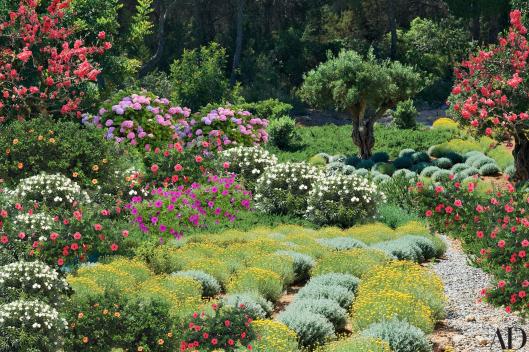 ○D. |
| 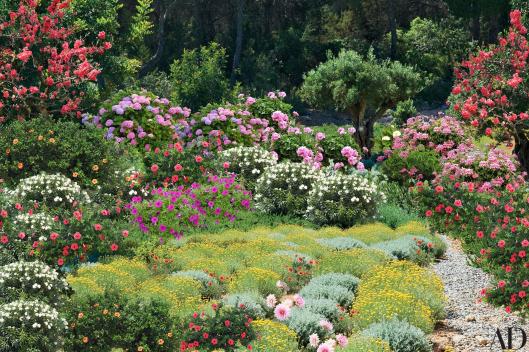 ○E. | |

9. When you are immersed in the scene of the following picture, please choose the scene with the best feeling according to your own preference [Single choice question] *

| 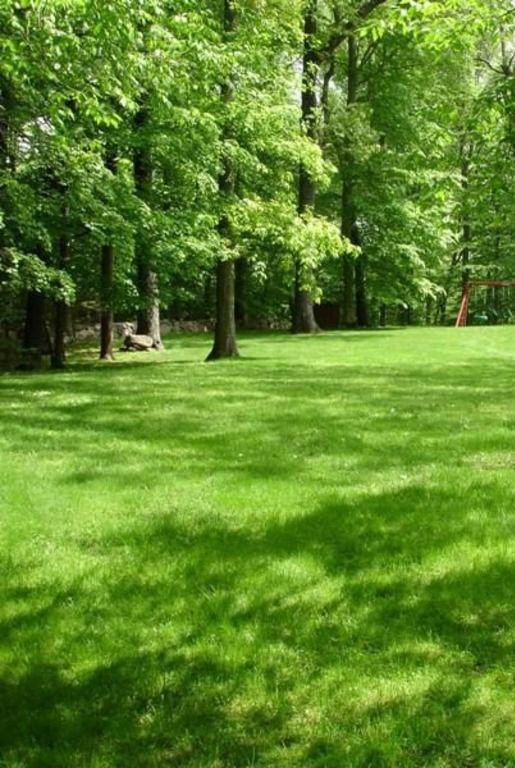 ○A. | 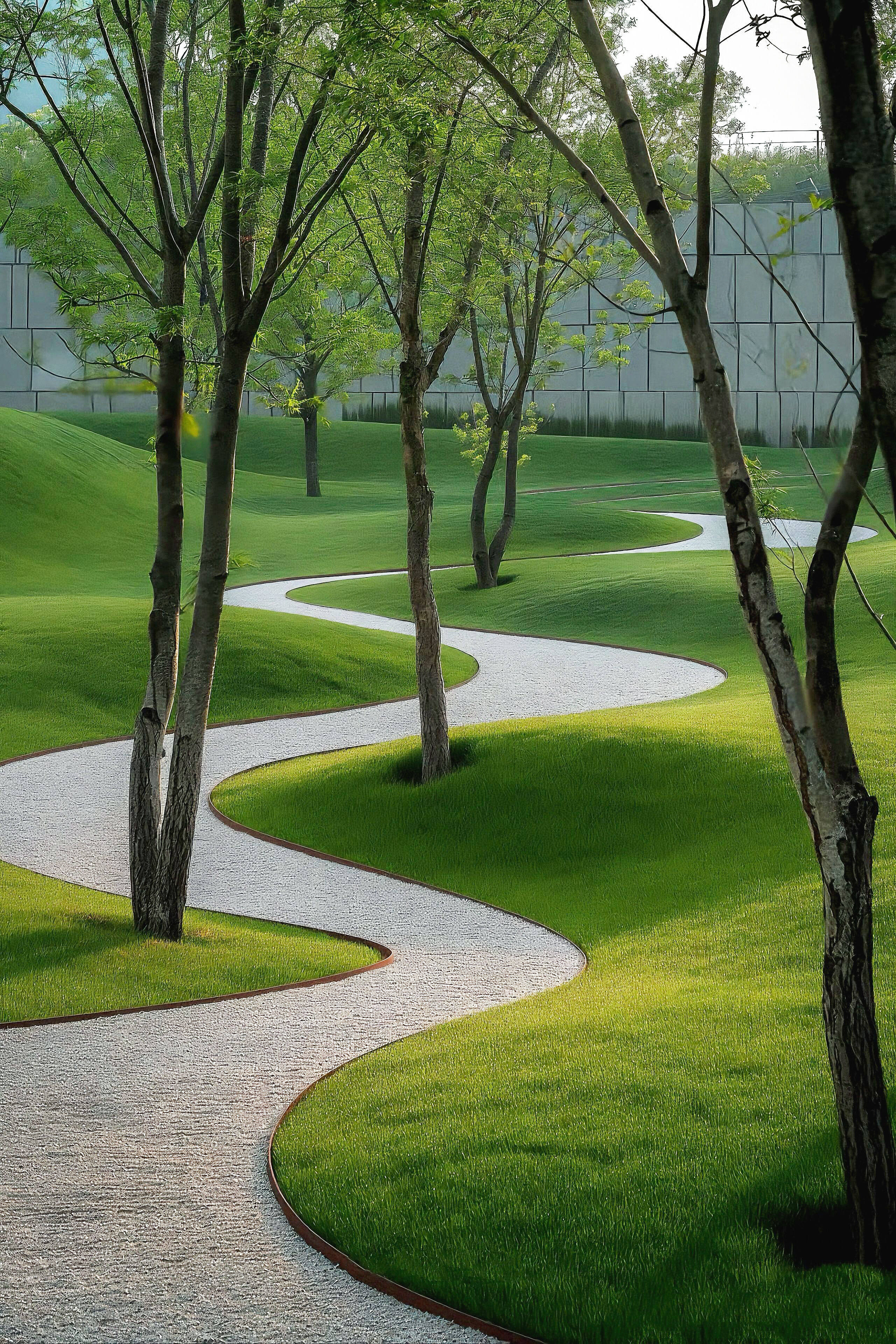 ○B. |
| --- | --- |
| 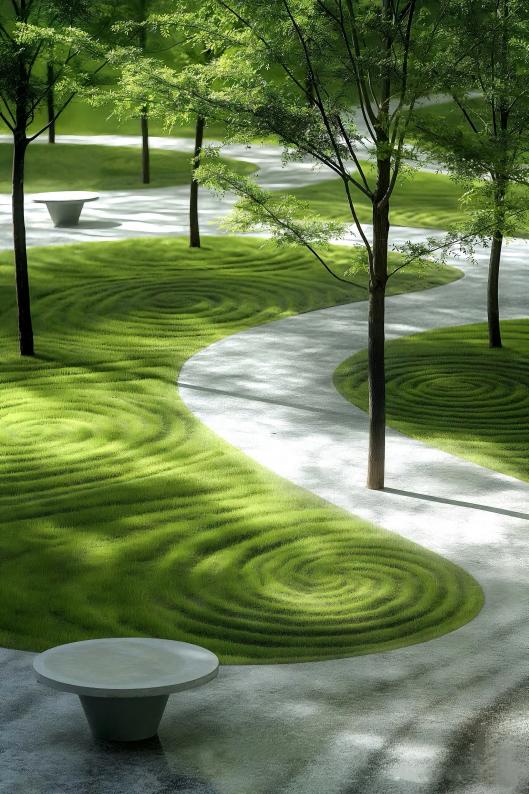 ○C. | 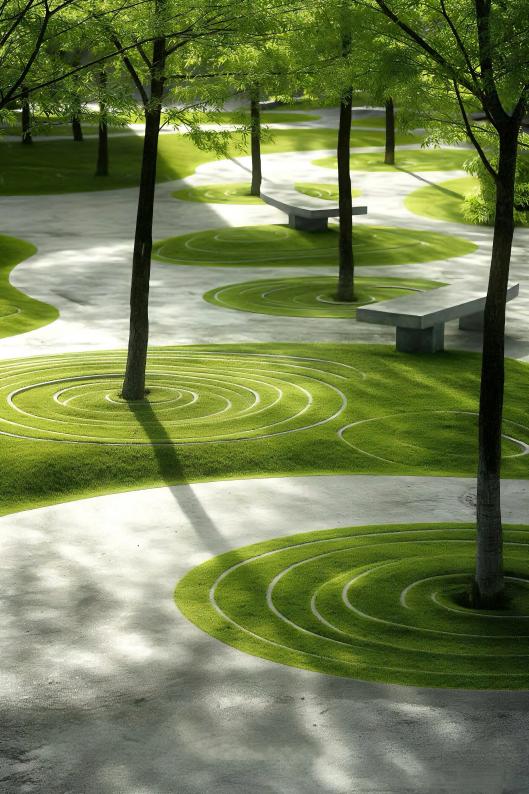 ○D. |
| 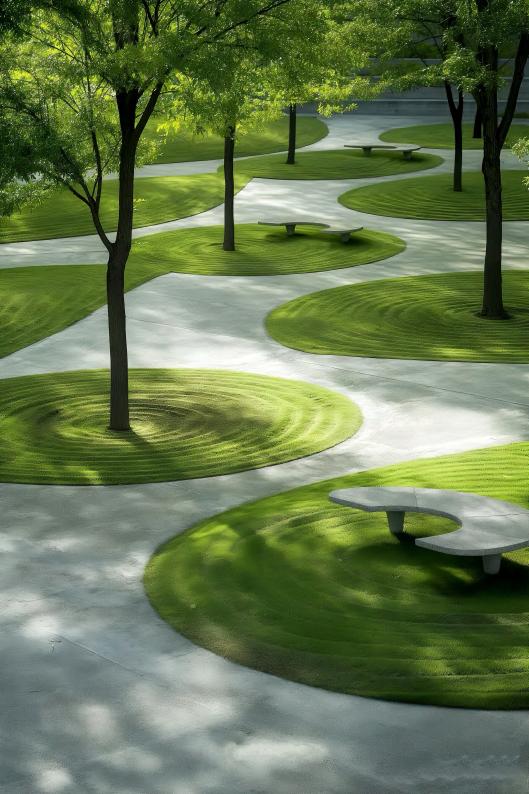 ○E. | |

10. When you are immersed in the scene of the following picture, please choose the scene with the best feeling according to your own preference [Single choice question] *

| 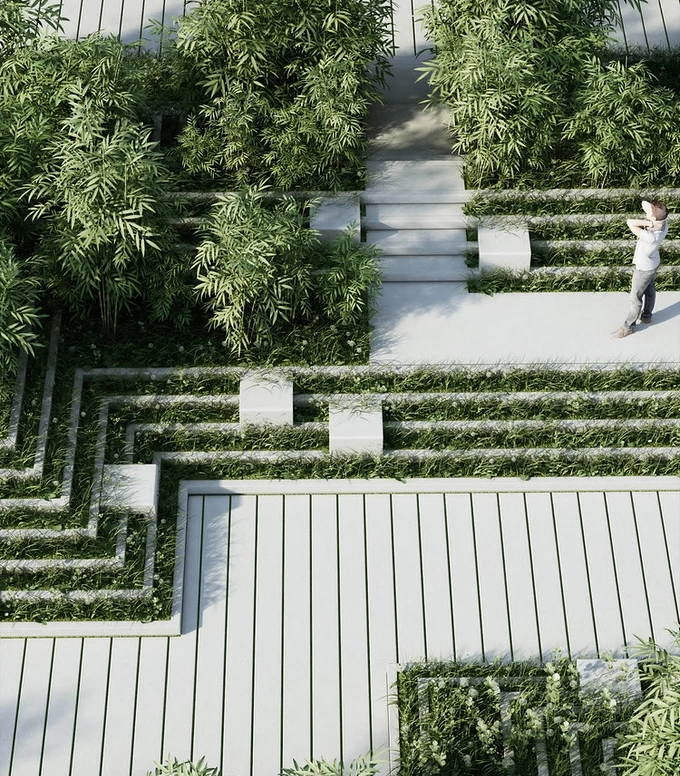 ○A.High altitude green space | 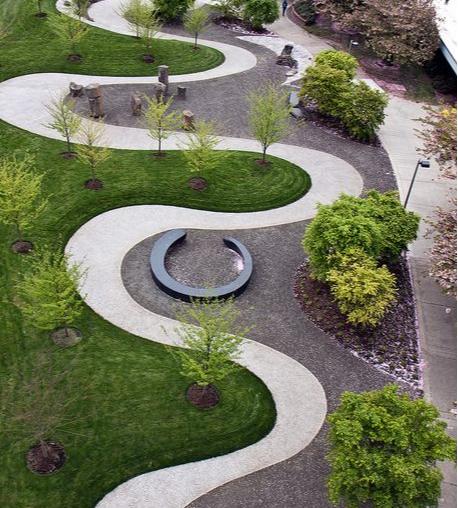 ○B.Curved green space |
| --- | --- |
| 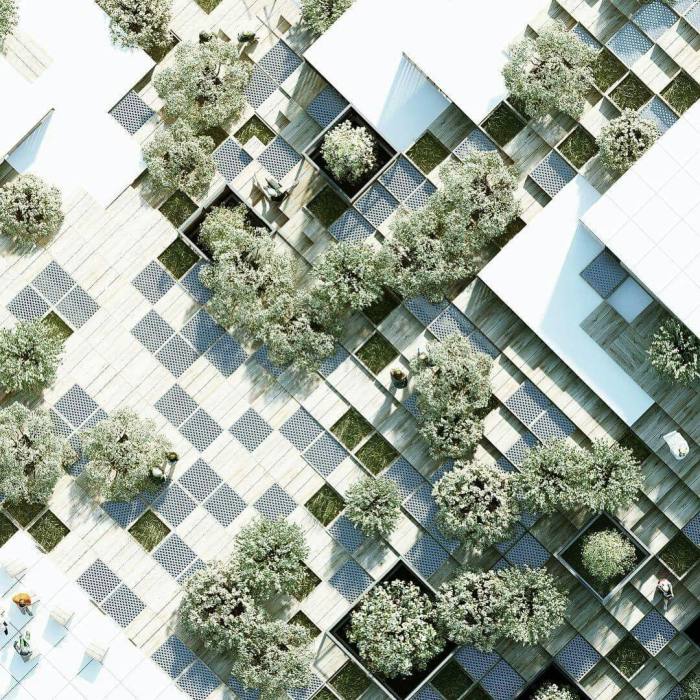 ○C.Rectangular green space | 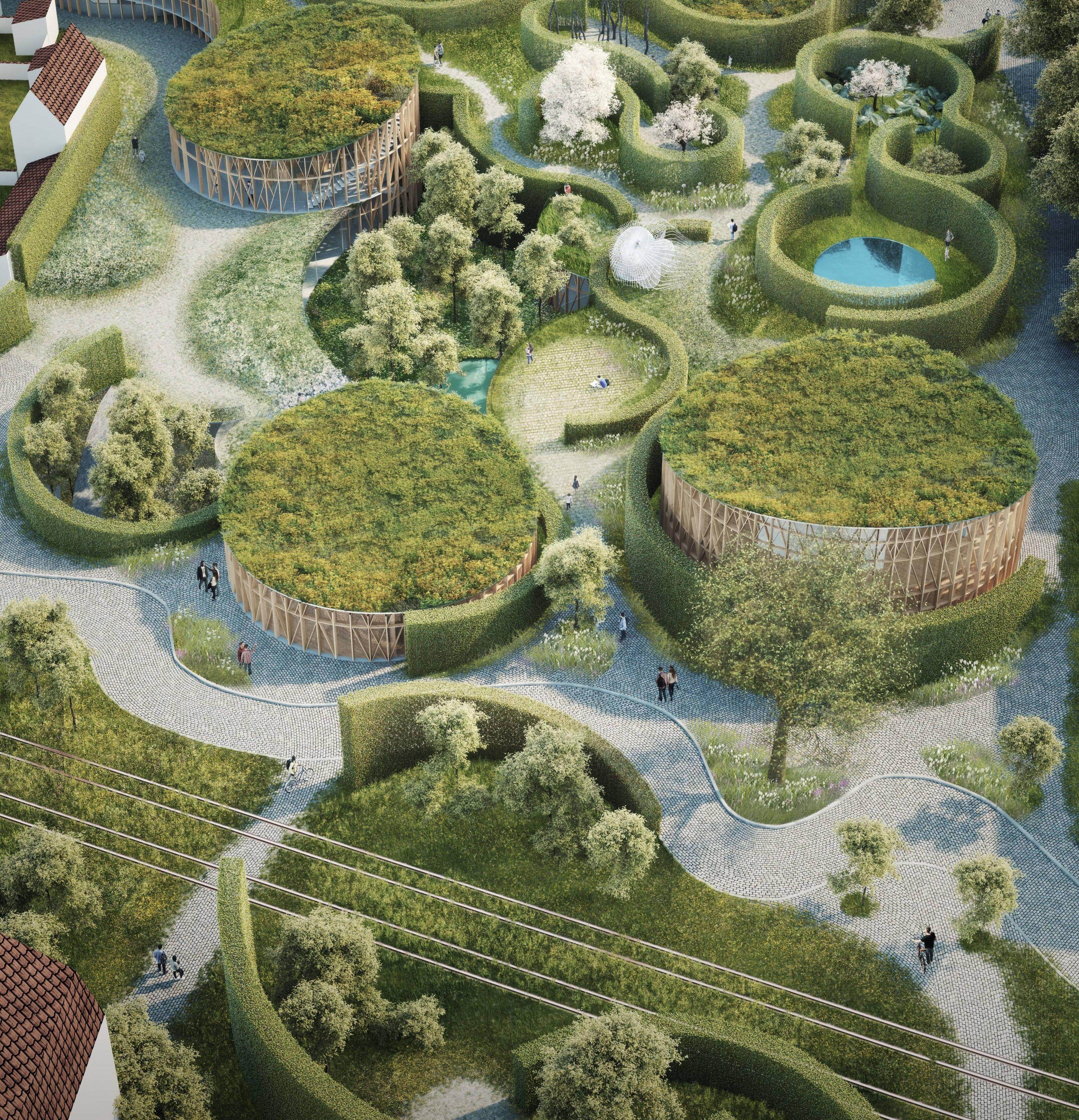 ○D.Circular green space |
| 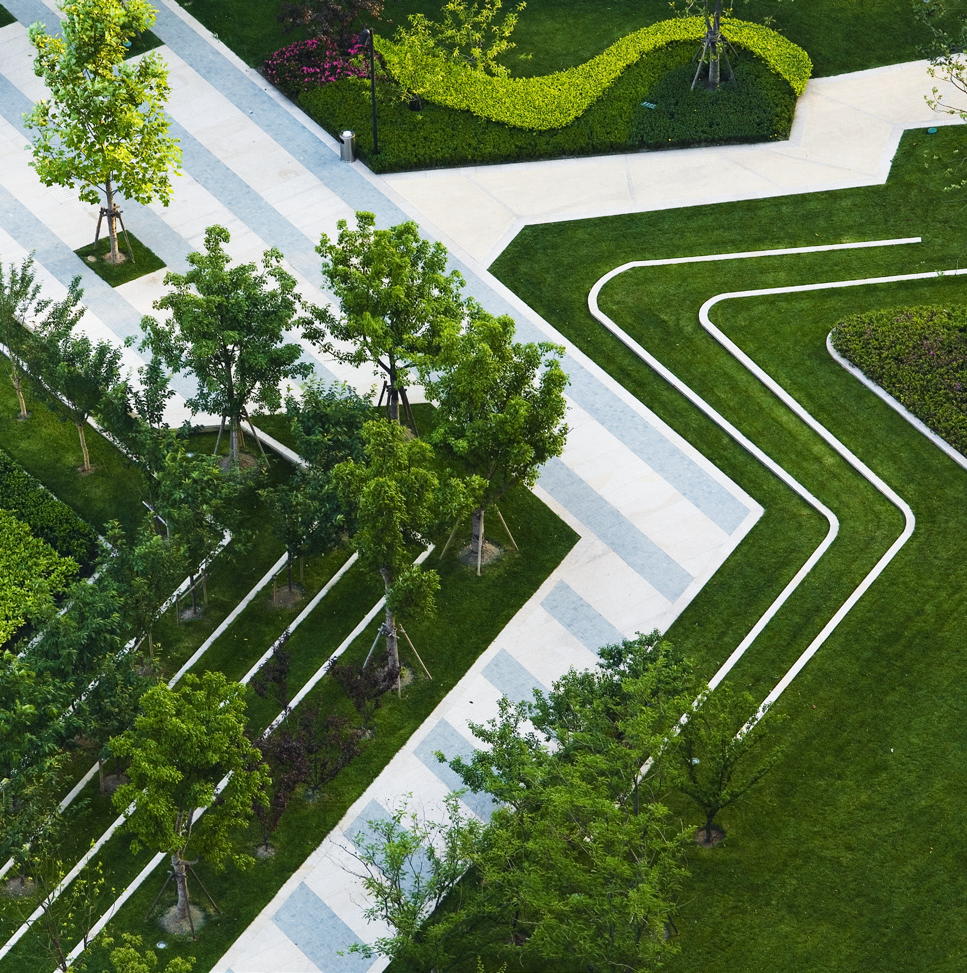 ○E.broken-line green space | |

**Part 2:** Rate the level of emotional excitement and relaxation that the above pictures have on you, and answer within the range of 0-10 (excitement refers to the positive emotional enhancement effect on individuals in the picture scene, which can be understood as pleasure; relaxation refers to the degree of emotional relaxation in the picture scene)

**① Green space richness**

11. This scene makes me feel uplifted


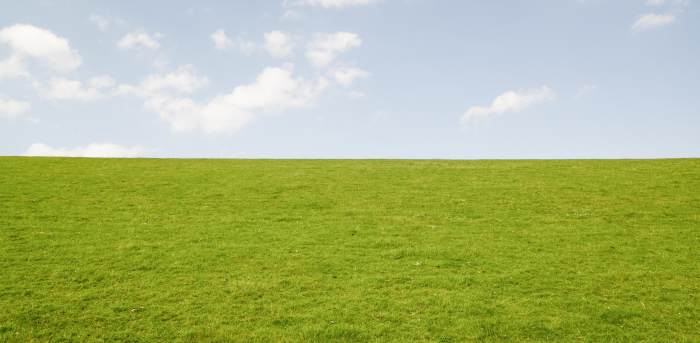


[Single choice question] *

| Very dissatisfied | ○1 | ○2 | ○3 | ○4 | ○5 | ○6 | ○7 | ○8 | ○9 | ○10 | Very satisfied |
| --- | --- | --- | --- | --- | --- | --- | --- | --- | --- | --- | --- |

12. This scene makes me feel relaxed


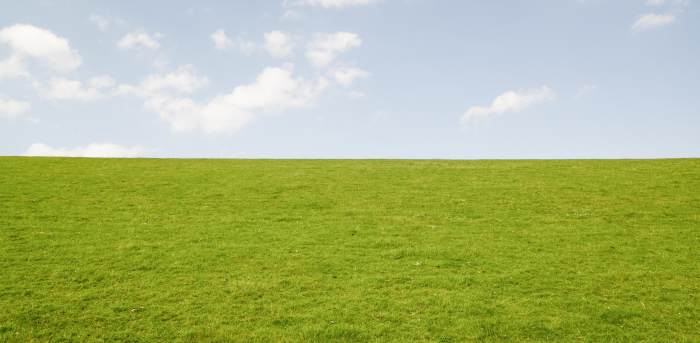


[Single choice question] *

| Very dissatisfied | ○1 | ○2 | ○3 | ○4 | ○5 | ○6 | ○7 | ○8 | ○9 | ○10 | Very satisfied |
| --- | --- | --- | --- | --- | --- | --- | --- | --- | --- | --- | --- |

1. This scene makes me feel uplifted


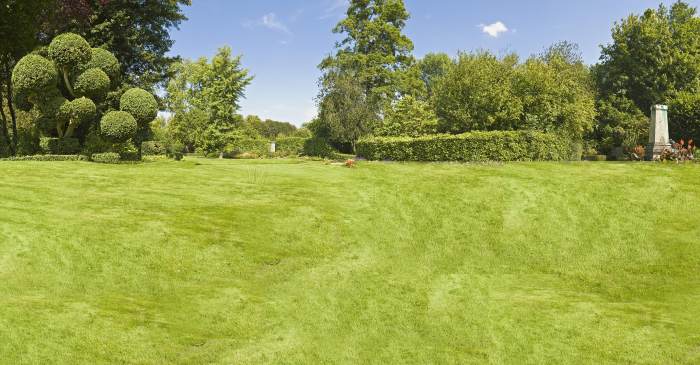


[Single choice question] *

| Very dissatisfied | ○1 | ○2 | ○3 | ○4 | ○5 | ○6 | ○7 | ○8 | ○9 | ○10 | Very satisfied |
| --- | --- | --- | --- | --- | --- | --- | --- | --- | --- | --- | --- |

1. This scene makes me feel relaxed


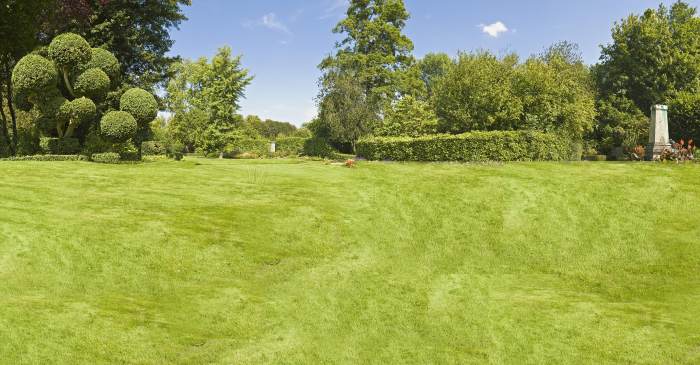


[Single choice question] *

| Very dissatisfied | ○1 | ○2 | ○3 | ○4 | ○5 | ○6 | ○7 | ○8 | ○9 | ○10 | Very satisfied |
| --- | --- | --- | --- | --- | --- | --- | --- | --- | --- | --- | --- |

1. This scene makes me feel uplifted


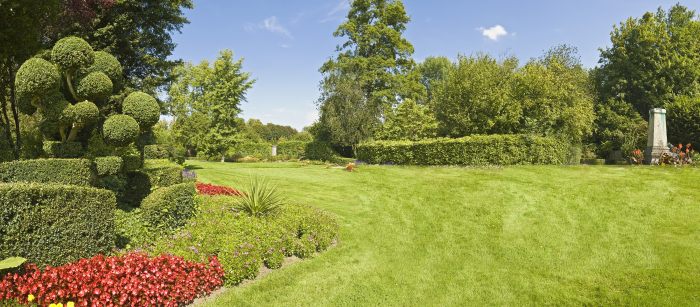


[Single choice question] *

| Very dissatisfied | ○1 | ○2 | ○3 | ○4 | ○5 | ○6 | ○7 | ○8 | ○9 | ○10 | Very satisfied |
| --- | --- | --- | --- | --- | --- | --- | --- | --- | --- | --- | --- |

16. This scene makes me feel relaxed


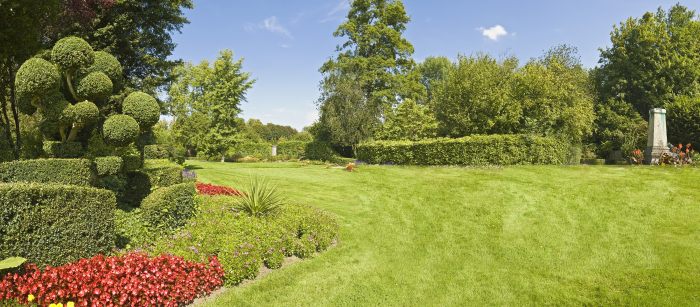


[Single choice question] *

| Very dissatisfied | ○1 | ○2 | ○3 | ○4 | ○5 | ○6 | ○7 | ○8 | ○9 | ○10 | Very satisfied |
| --- | --- | --- | --- | --- | --- | --- | --- | --- | --- | --- | --- |

17. This scene makes me feel uplifted


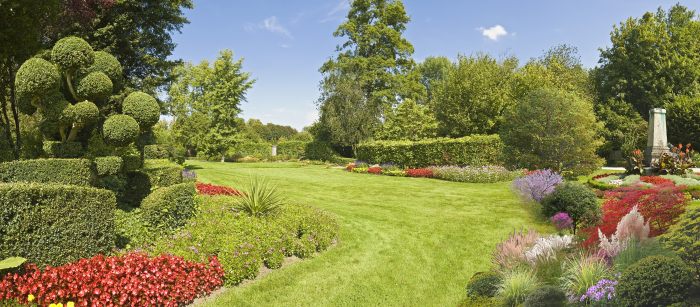


[Single choice question] *

| Very dissatisfied | ○1 | ○2 | ○3 | ○4 | ○5 | ○6 | ○7 | ○8 | ○9 | ○10 | Very satisfied |
| --- | --- | --- | --- | --- | --- | --- | --- | --- | --- | --- | --- |

18. This scene makes me feel relaxed


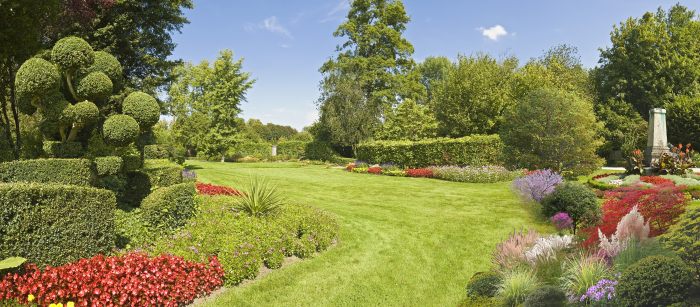


[Single choice question] *

| Very dissatisfied | ○1 | ○2 | ○3 | ○4 | ○5 | ○6 | ○7 | ○8 | ○9 | ○10 | Very satisfied |
| --- | --- | --- | --- | --- | --- | --- | --- | --- | --- | --- | --- |

19. This scene makes me feel uplifted


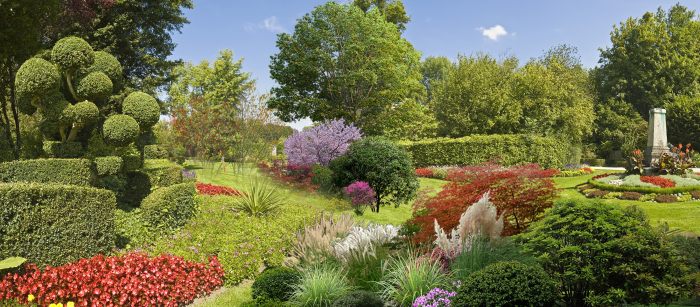


[Single choice question] *

| Very dissatisfied | ○1 | ○2 | ○3 | ○4 | ○5 | ○6 | ○7 | ○8 | ○9 | ○10 | Very satisfied |
| --- | --- | --- | --- | --- | --- | --- | --- | --- | --- | --- | --- |

20. This scene makes me feel relaxed


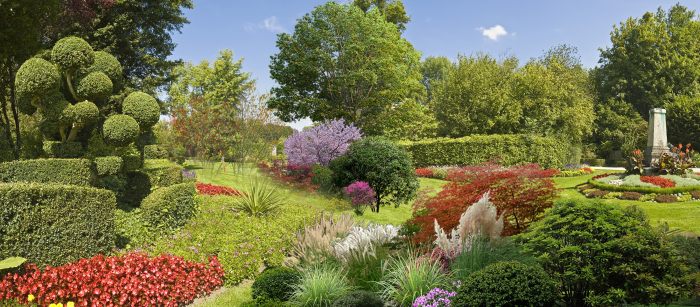


[Single choice question] *

| Very dissatisfied | ○1 | ○2 | ○3 | ○4 | ○5 | ○6 | ○7 | ○8 | ○9 | ○10 | Very satisfied |
| --- | --- | --- | --- | --- | --- | --- | --- | --- | --- | --- | --- |

**② Combination ratio of flowers and plants**

21. This scene makes me feel uplifted


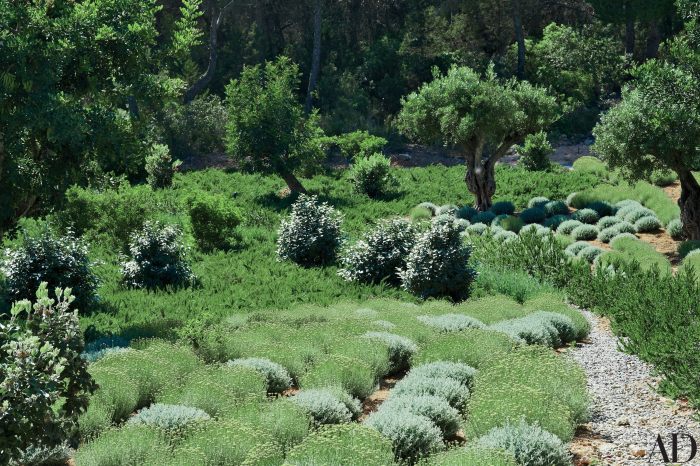


[Single choice question] *

| Very dissatisfied | ○1 | ○2 | ○3 | ○4 | ○5 | ○6 | ○7 | ○8 | ○9 | ○10 | Very satisfied |
| --- | --- | --- | --- | --- | --- | --- | --- | --- | --- | --- | --- |

22. This scene makes me feel relaxed


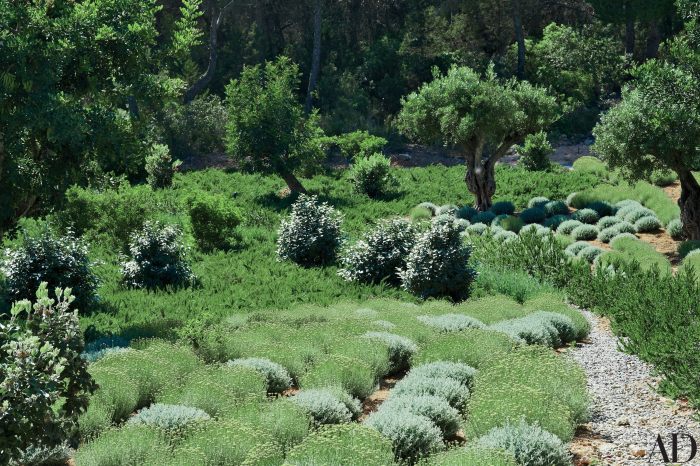


[Single choice question] *

| Very dissatisfied | ○1 | ○2 | ○3 | ○4 | ○5 | ○6 | ○7 | ○8 | ○9 | ○10 | Very satisfied |
| --- | --- | --- | --- | --- | --- | --- | --- | --- | --- | --- | --- |

23. This scene makes me feel uplifted


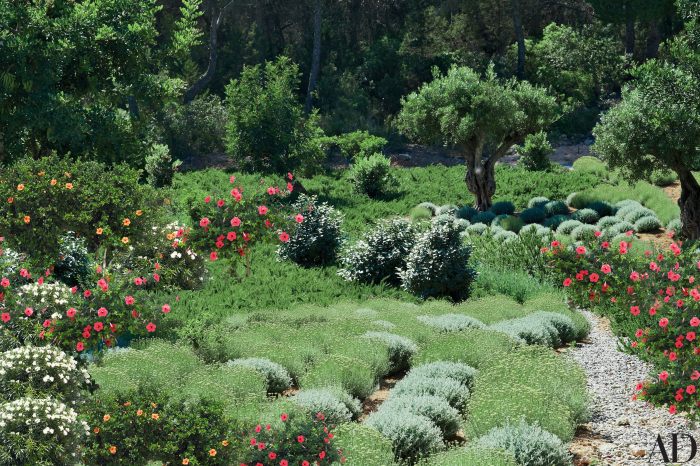


[Single choice question] *

| Very dissatisfied | ○1 | ○2 | ○3 | ○4 | ○5 | ○6 | ○7 | ○8 | ○9 | ○10 | Very satisfied |
| --- | --- | --- | --- | --- | --- | --- | --- | --- | --- | --- | --- |

24. This scene makes me feel relaxed


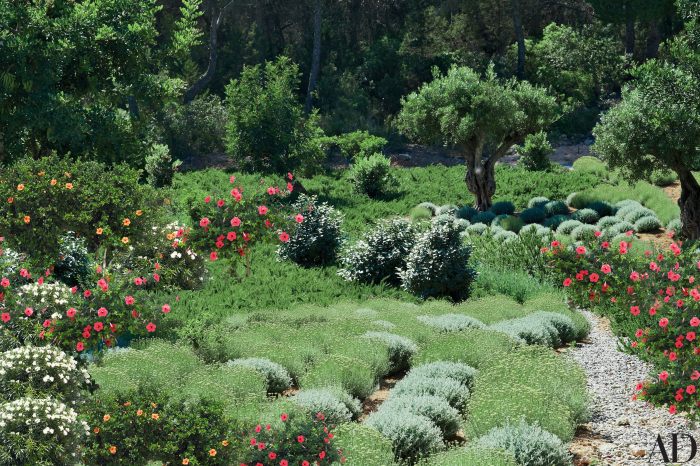


[Single choice question] *

| Very dissatisfied | ○1 | ○2 | ○3 | ○4 | ○5 | ○6 | ○7 | ○8 | ○9 | ○10 | Very satisfied |
| --- | --- | --- | --- | --- | --- | --- | --- | --- | --- | --- | --- |

25. This scene makes me feel uplifted


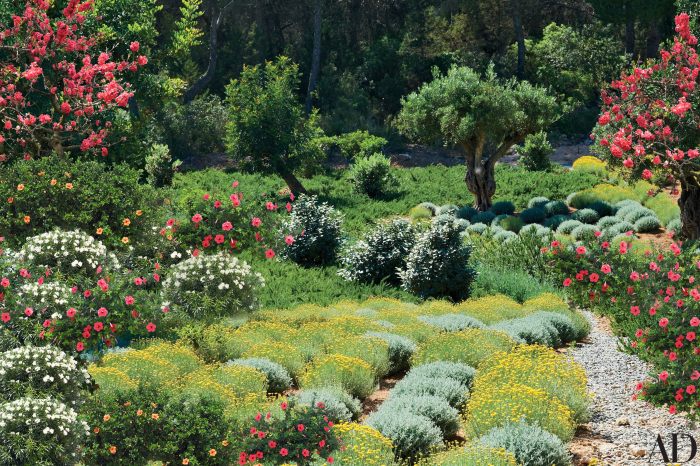


[Single choice question] *

| Very dissatisfied | ○1 | ○2 | ○3 | ○4 | ○5 | ○6 | ○7 | ○8 | ○9 | ○10 | Very satisfied |
| --- | --- | --- | --- | --- | --- | --- | --- | --- | --- | --- | --- |

1. This scene makes me feel relaxed


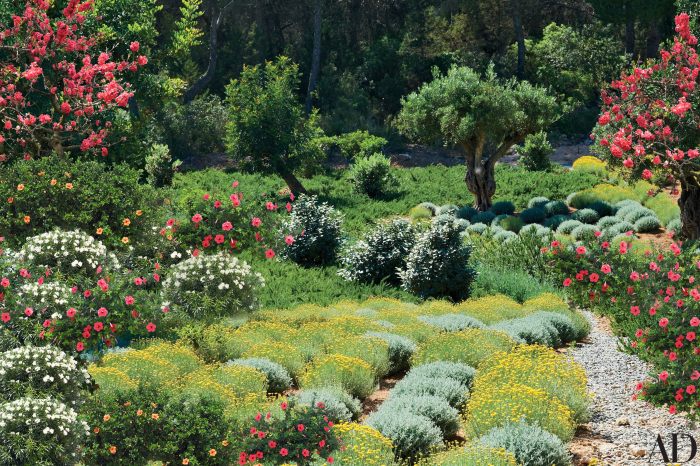


[Single choice question] *

| Very dissatisfied | ○1 | ○2 | ○3 | ○4 | ○5 | ○6 | ○7 | ○8 | ○9 | ○10 | Very satisfied |
| --- | --- | --- | --- | --- | --- | --- | --- | --- | --- | --- | --- |

27. This scene makes me feel uplifted


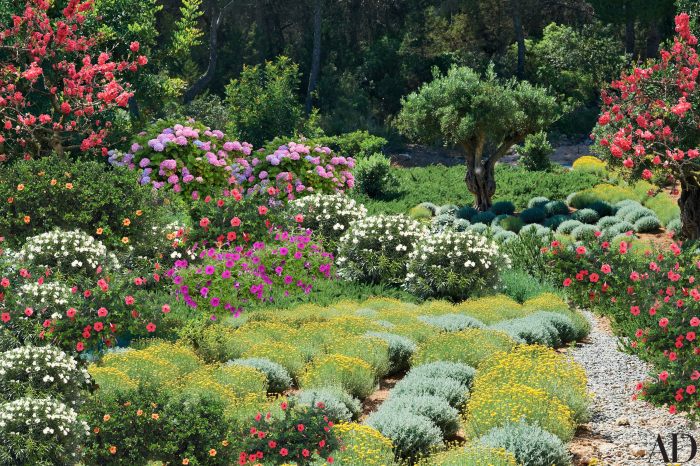


[Single choice question] *

| Very dissatisfied | ○1 | ○2 | ○3 | ○4 | ○5 | ○6 | ○7 | ○8 | ○9 | ○10 | Very satisfied |
| --- | --- | --- | --- | --- | --- | --- | --- | --- | --- | --- | --- |

28. This scene makes me feel relaxed


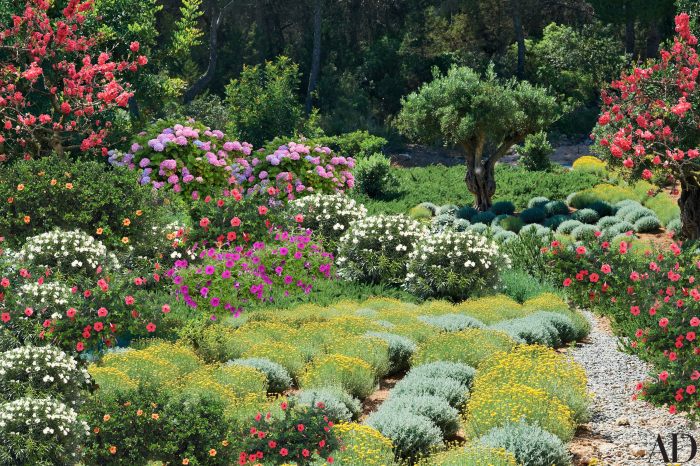


[Single choice question] *

| Very dissatisfied | ○1 | ○2 | ○3 | ○4 | ○5 | ○6 | ○7 | ○8 | ○9 | ○10 | Very satisfied |
| --- | --- | --- | --- | --- | --- | --- | --- | --- | --- | --- | --- |

29. This scene makes me feel uplifted


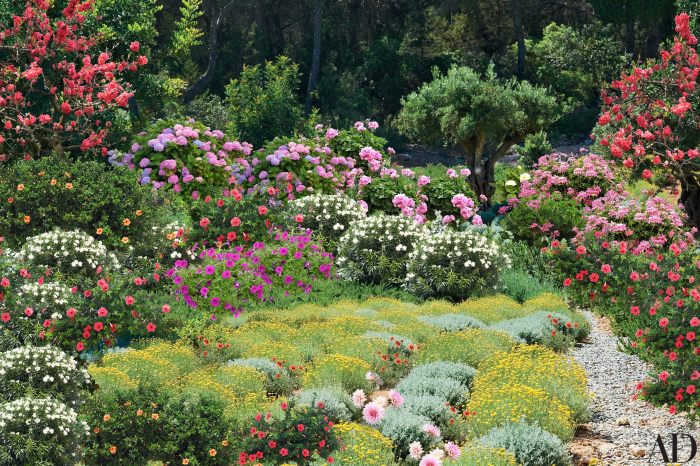


[Single choice question] *

| Very dissatisfied | ○1 | ○2 | ○3 | ○4 | ○5 | ○6 | ○7 | ○8 | ○9 | ○10 | Very satisfied |
| --- | --- | --- | --- | --- | --- | --- | --- | --- | --- | --- | --- |

30. This scene makes me feel relaxed


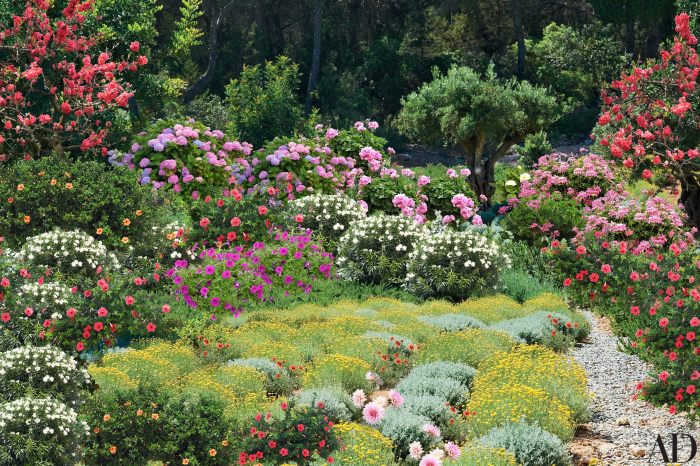


[Single choice question] *

| Very dissatisfied | ○1 | ○2 | ○3 | ○4 | ○5 | ○6 | ○7 | ○8 | ○9 | ○10 | Very satisfied |
| --- | --- | --- | --- | --- | --- | --- | --- | --- | --- | --- | --- |

**③ Green connectivity**

31. This scene makes me feel uplifted


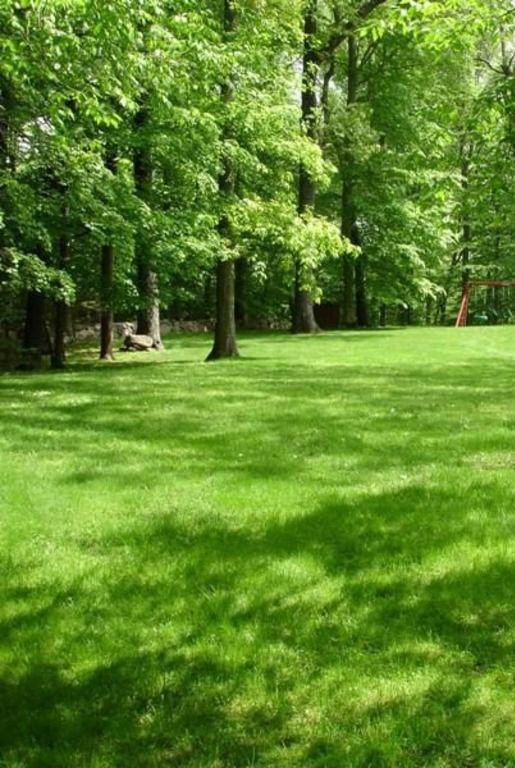


[Single choice question] *

| Very dissatisfied | ○1 | ○2 | ○3 | ○4 | ○5 | ○6 | ○7 | ○8 | ○9 | ○10 | Very satisfied |
| --- | --- | --- | --- | --- | --- | --- | --- | --- | --- | --- | --- |

32. This scene makes me feel relaxed


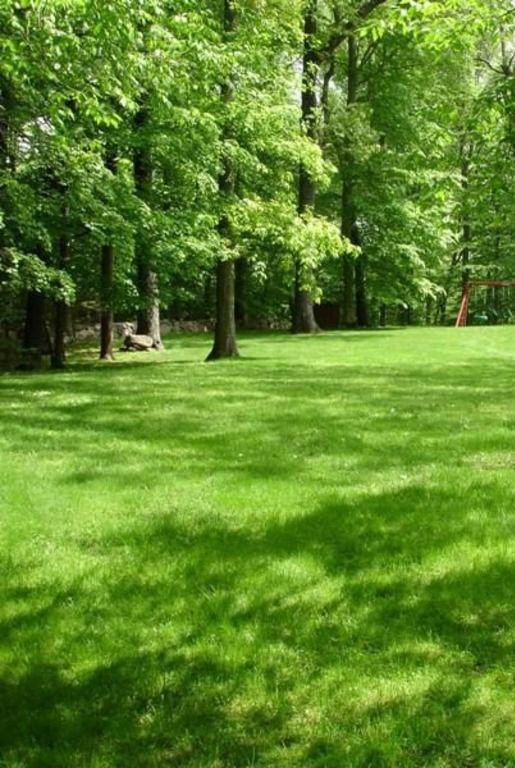


[Single choice question] *

| Very dissatisfied | ○1 | ○2 | ○3 | ○4 | ○5 | ○6 | ○7 | ○8 | ○9 | ○10 | Very satisfied |
| --- | --- | --- | --- | --- | --- | --- | --- | --- | --- | --- | --- |

33. This scene makes me feel uplifted


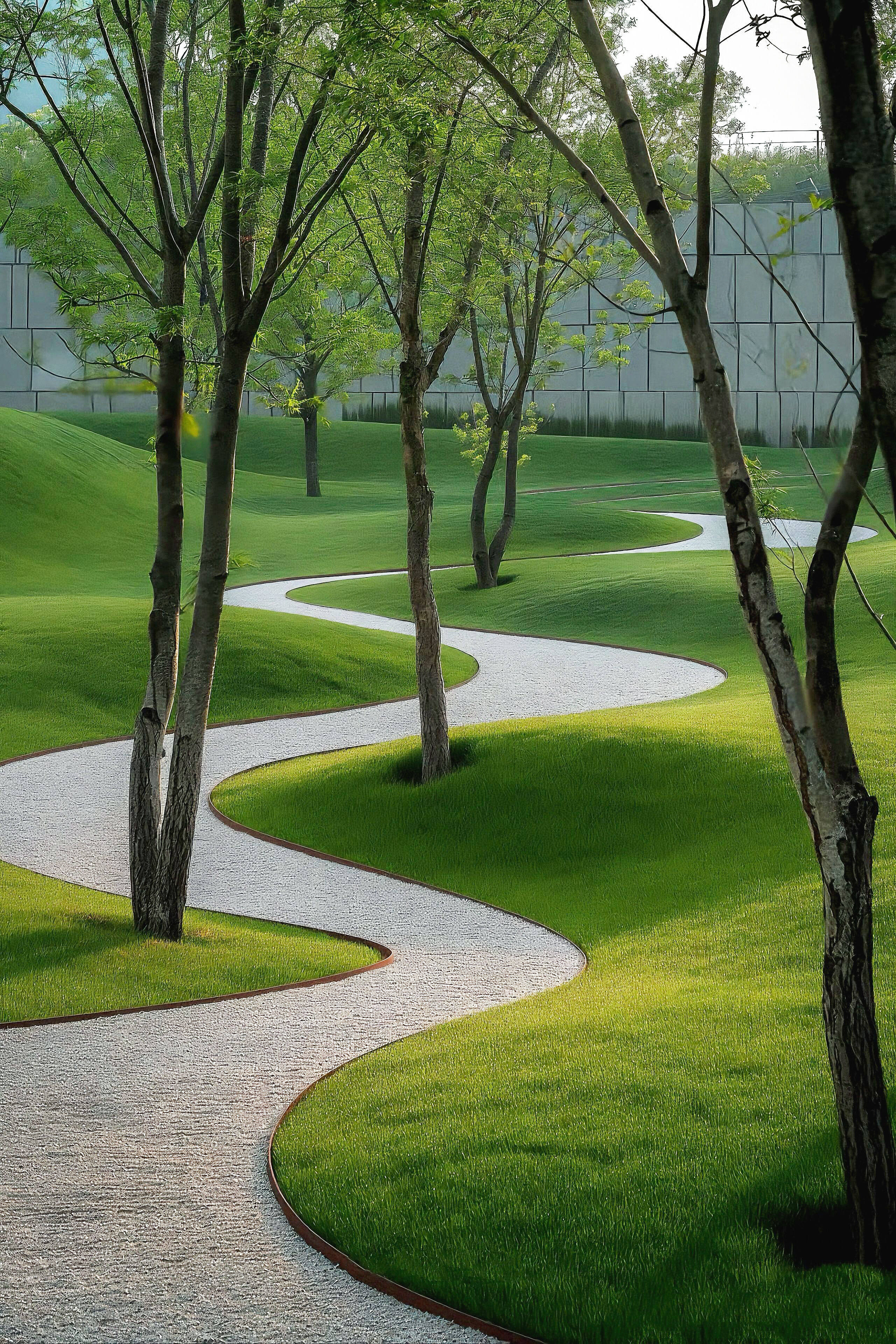


[Single choice question] *

| Very dissatisfied | ○1 | ○2 | ○3 | ○4 | ○5 | ○6 | ○7 | ○8 | ○9 | ○10 | Very satisfied |
| --- | --- | --- | --- | --- | --- | --- | --- | --- | --- | --- | --- |

34. This scene makes me feel relaxed


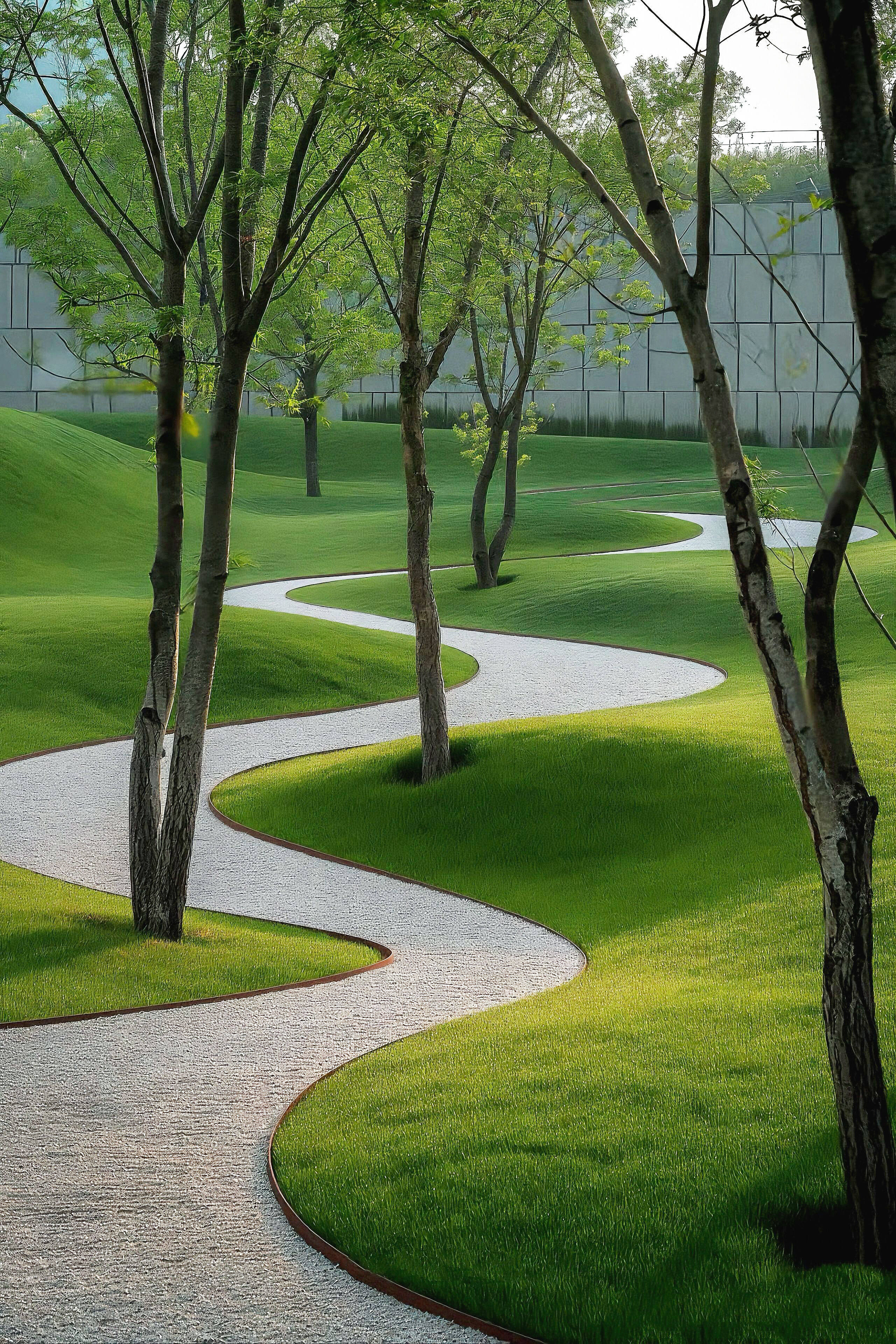


[Single choice question] *

| Very dissatisfied | ○1 | ○2 | ○3 | ○4 | ○5 | ○6 | ○7 | ○8 | ○9 | ○10 | Very satisfied |
| --- | --- | --- | --- | --- | --- | --- | --- | --- | --- | --- | --- |

35. This scene makes me feel uplifted


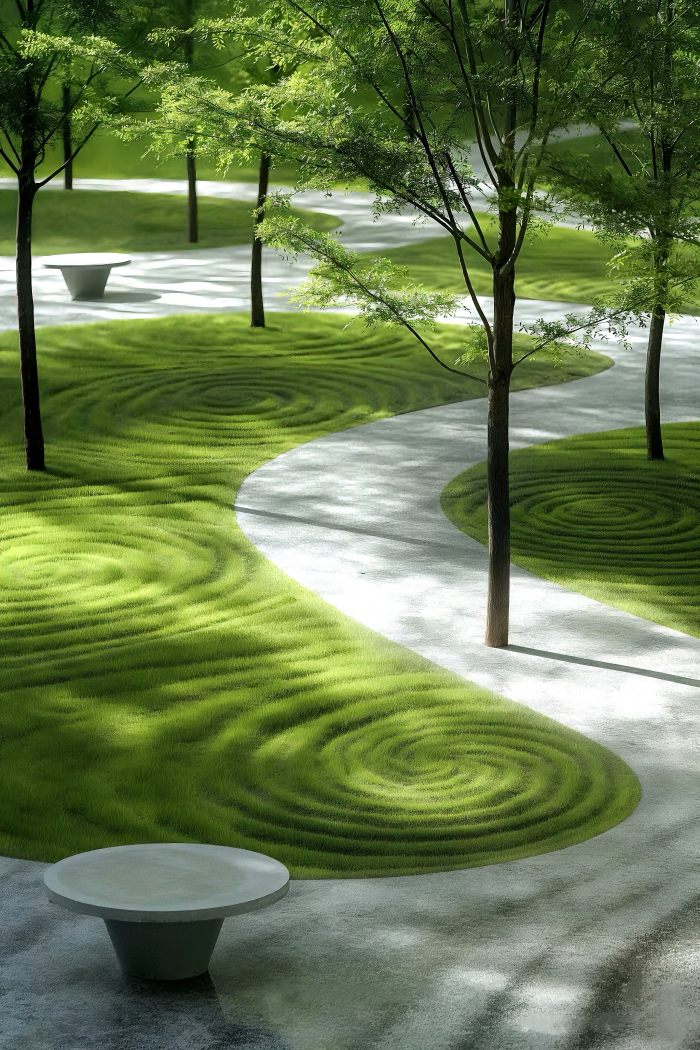


[Single choice question] *

| Very dissatisfied | ○1 | ○2 | ○3 | ○4 | ○5 | ○6 | ○7 | ○8 | ○9 | ○10 | Very satisfied |
| --- | --- | --- | --- | --- | --- | --- | --- | --- | --- | --- | --- |

36. This scene makes me feel relaxed


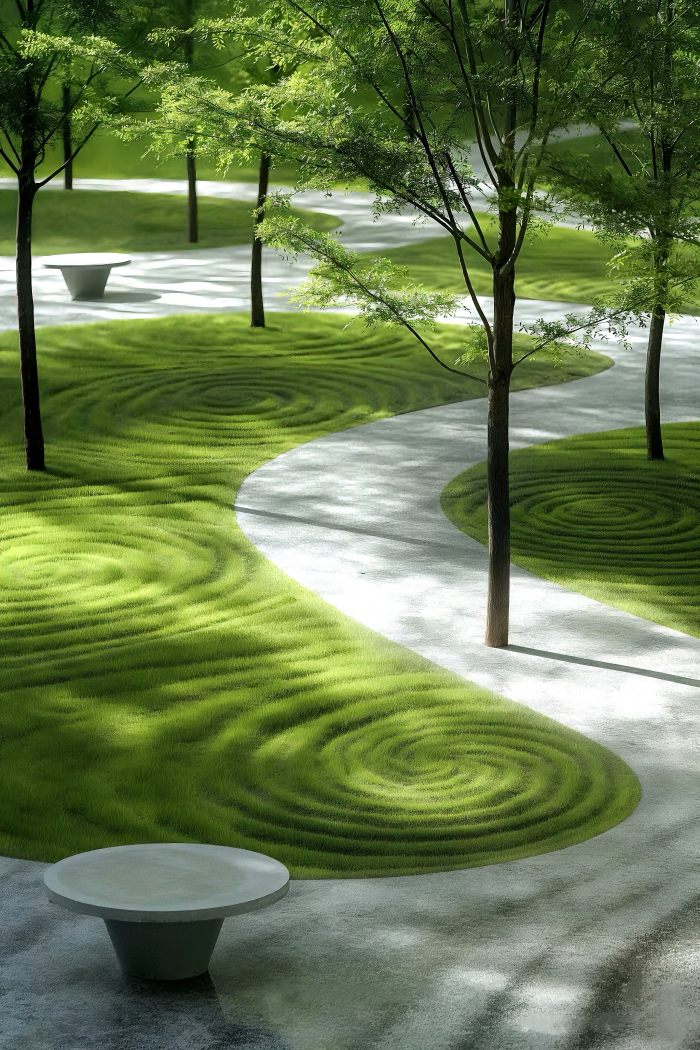


[Single choice question] *

| Very dissatisfied | ○1 | ○2 | ○3 | ○4 | ○5 | ○6 | ○7 | ○8 | ○9 | ○10 | Very satisfied |
| --- | --- | --- | --- | --- | --- | --- | --- | --- | --- | --- | --- |

37. This scene makes me feel uplifted


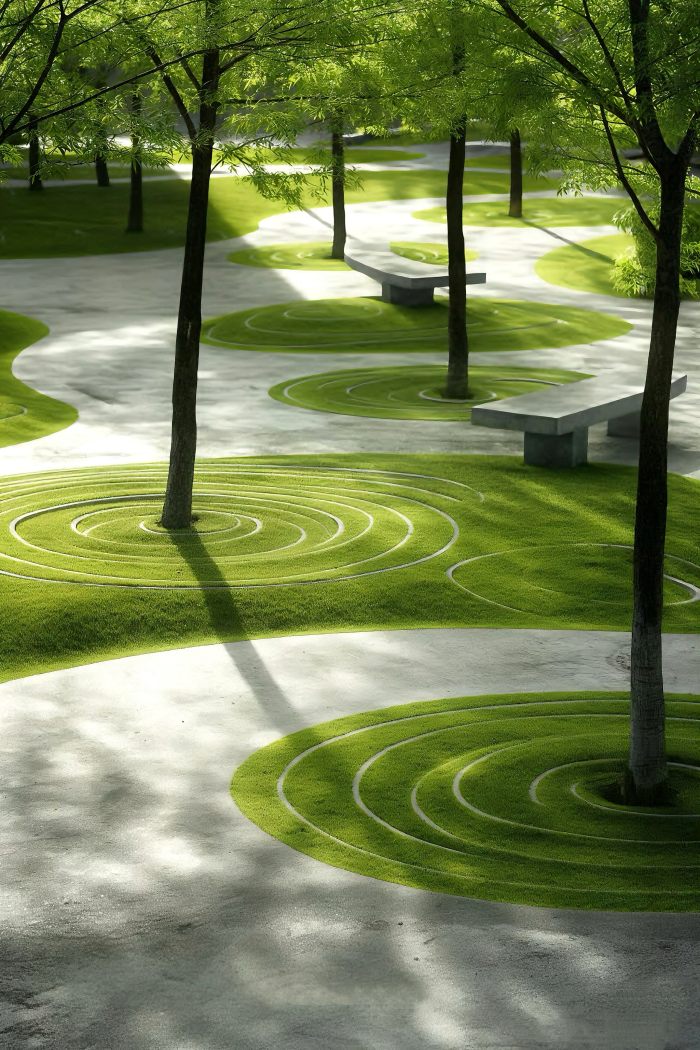


[Single choice question] *

| Very dissatisfied | ○1 | ○2 | ○3 | ○4 | ○5 | ○6 | ○7 | ○8 | ○9 | ○10 | Very satisfied |
| --- | --- | --- | --- | --- | --- | --- | --- | --- | --- | --- | --- |

38. This scene makes me feel relaxed


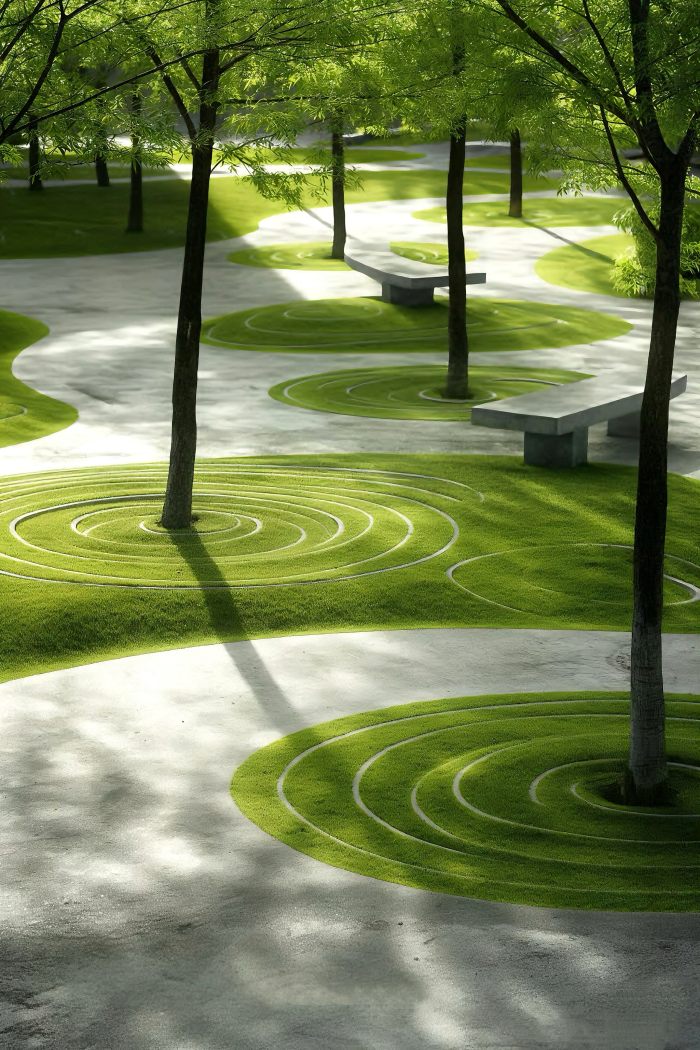


[Single choice question] *

| Very dissatisfied | ○1 | ○2 | ○3 | ○4 | ○5 | ○6 | ○7 | ○8 | ○9 | ○10 | Very satisfied |
| --- | --- | --- | --- | --- | --- | --- | --- | --- | --- | --- | --- |

39. This scene makes me feel uplifted


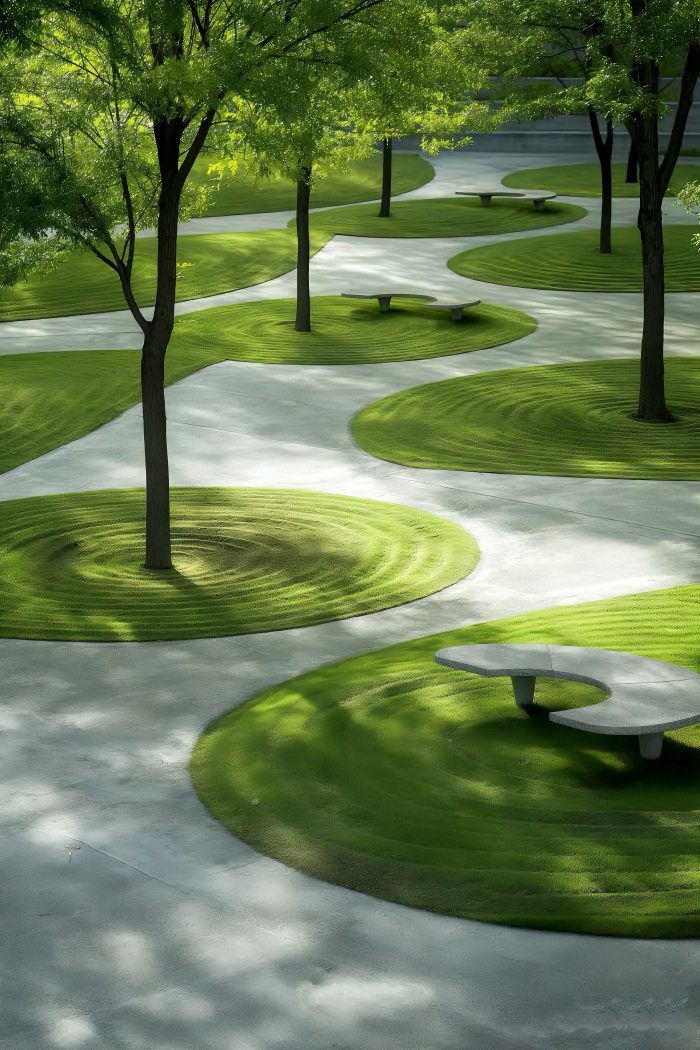


[Single choice question] *

| Very dissatisfied | ○1 | ○2 | ○3 | ○4 | ○5 | ○6 | ○7 | ○8 | ○9 | ○10 | Very satisfied |
| --- | --- | --- | --- | --- | --- | --- | --- | --- | --- | --- | --- |

40. This scene makes me feel relaxed


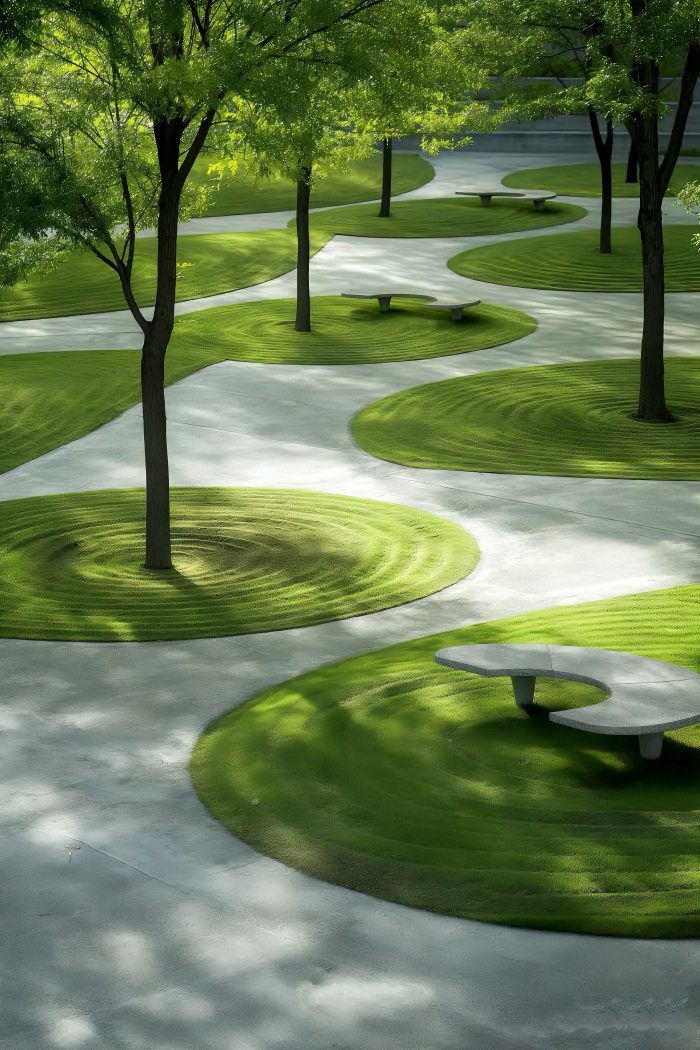


[Single choice question] *

| Very dissatisfied | ○1 | ○2 | ○3 | ○4 | ○5 | ○6 | ○7 | ○8 | ○9 | ○10 | Very satisfied |
| --- | --- | --- | --- | --- | --- | --- | --- | --- | --- | --- | --- |

**④ Green space form**

41. This scene makes me feel uplifted


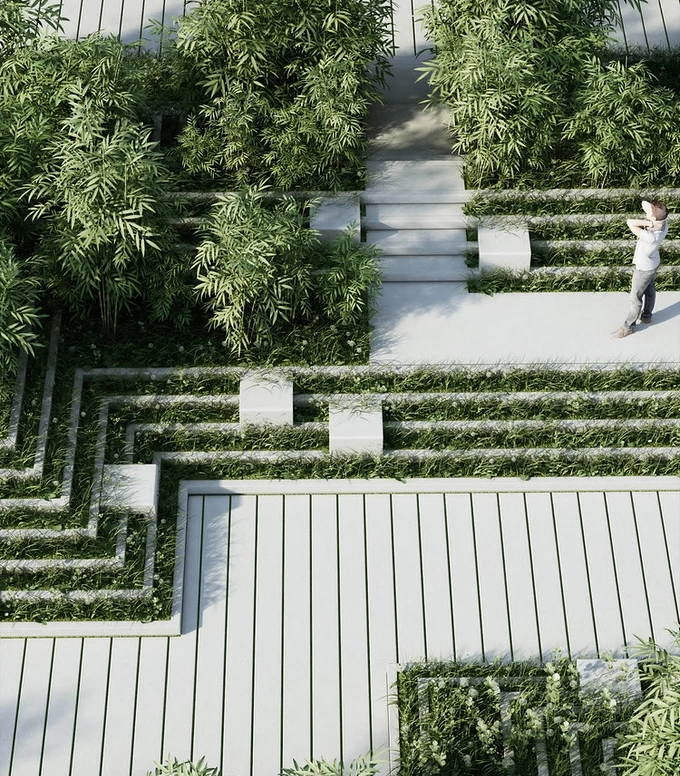


[Single choice question] *

| Very dissatisfied | ○1 | ○2 | ○3 | ○4 | ○5 | ○6 | ○7 | ○8 | ○9 | ○10 | Very satisfied |
| --- | --- | --- | --- | --- | --- | --- | --- | --- | --- | --- | --- |

42. This scene makes me feel relaxed


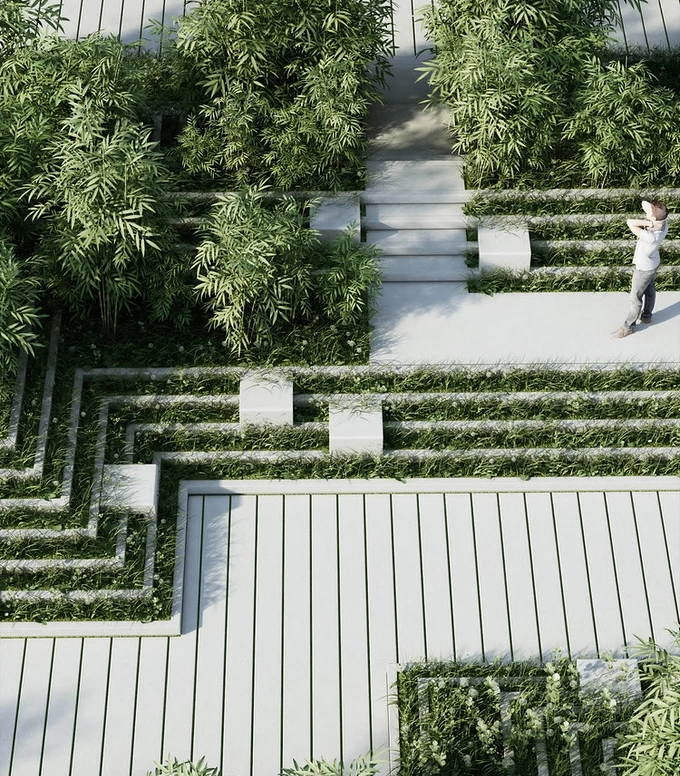


[Single choice question] *

| Very dissatisfied | ○1 | ○2 | ○3 | ○4 | ○5 | ○6 | ○7 | ○8 | ○9 | ○10 | Very satisfied |
| --- | --- | --- | --- | --- | --- | --- | --- | --- | --- | --- | --- |

43. This scene makes me feel uplifted


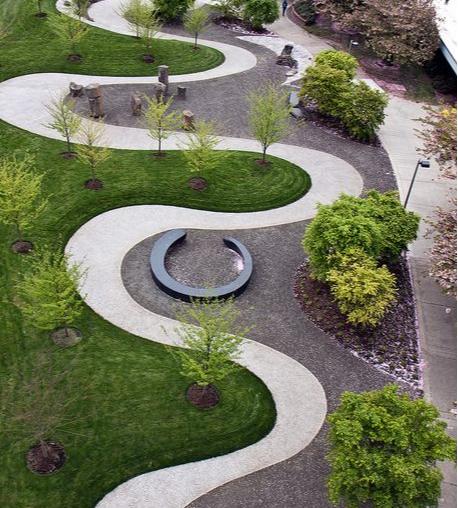


[Single choice question] *

| Very dissatisfied | ○1 | ○2 | ○3 | ○4 | ○5 | ○6 | ○7 | ○8 | ○9 | ○10 | Very satisfied |
| --- | --- | --- | --- | --- | --- | --- | --- | --- | --- | --- | --- |

44. This scene makes me feel relaxed


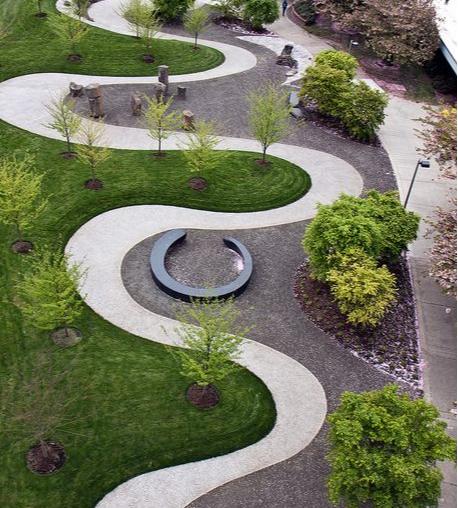


[Single choice question] *

| Very dissatisfied | ○1 | ○2 | ○3 | ○4 | ○5 | ○6 | ○7 | ○8 | ○9 | ○10 | Very satisfied |
| --- | --- | --- | --- | --- | --- | --- | --- | --- | --- | --- | --- |

45. This scene makes me feel uplifted


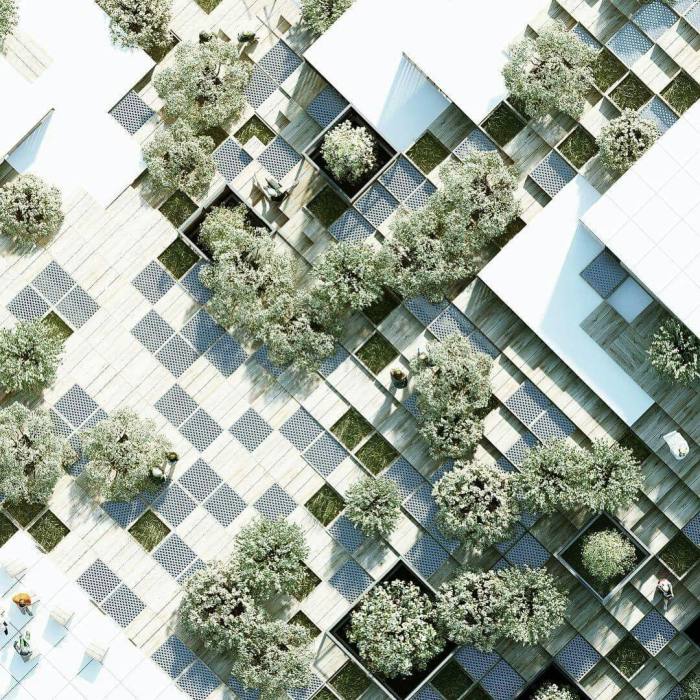


[Single choice question] *

| Very dissatisfied | ○1 | ○2 | ○3 | ○4 | ○5 | ○6 | ○7 | ○8 | ○9 | ○10 | Very satisfied |
| --- | --- | --- | --- | --- | --- | --- | --- | --- | --- | --- | --- |

46. This scene makes me feel relaxed


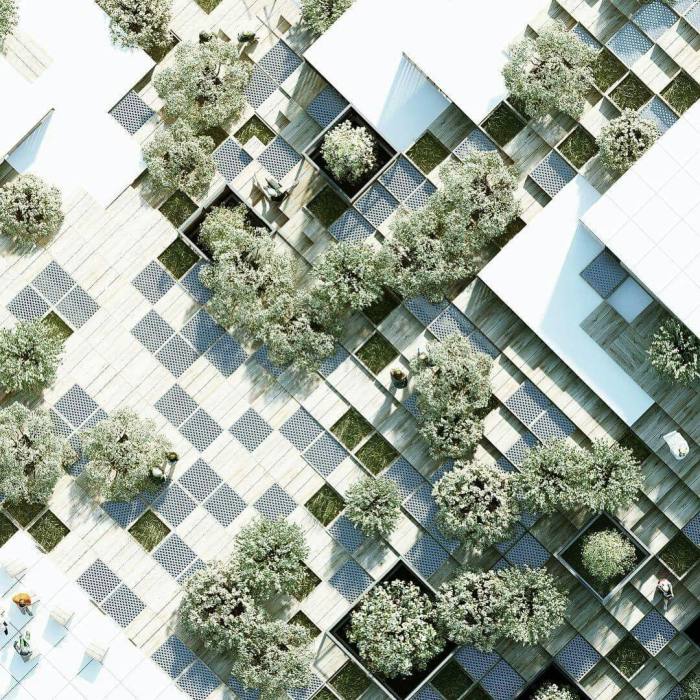


[Single choice question] *

| Very dissatisfied | ○1 | ○2 | ○3 | ○4 | ○5 | ○6 | ○7 | ○8 | ○9 | ○10 | Very satisfied |
| --- | --- | --- | --- | --- | --- | --- | --- | --- | --- | --- | --- |

47. This scene makes me feel uplifted


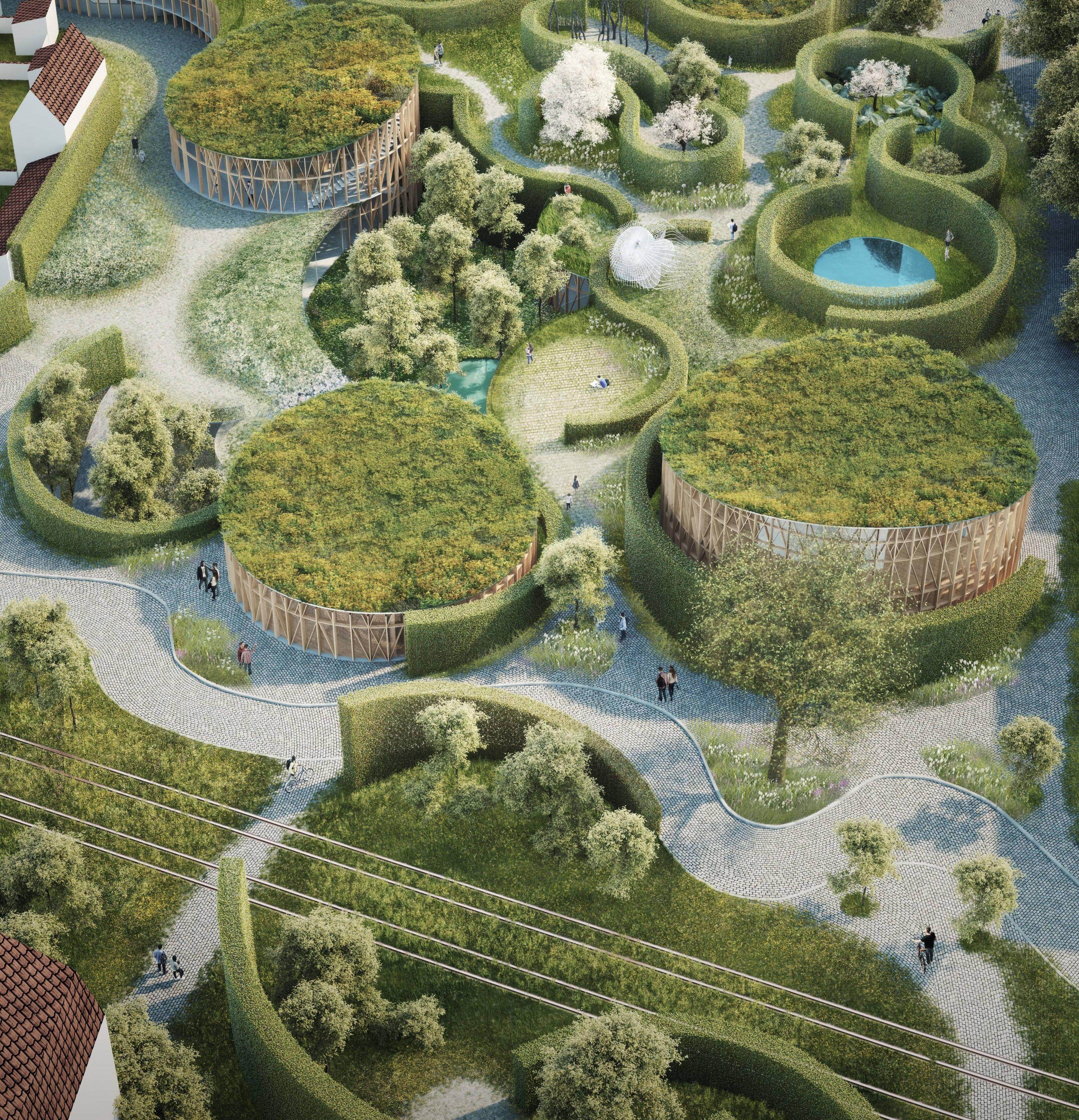


[Single choice question] *

| Very dissatisfied | ○1 | ○2 | ○3 | ○4 | ○5 | ○6 | ○7 | ○8 | ○9 | ○10 | Very satisfied |
| --- | --- | --- | --- | --- | --- | --- | --- | --- | --- | --- | --- |

48. This scene makes me feel relaxed


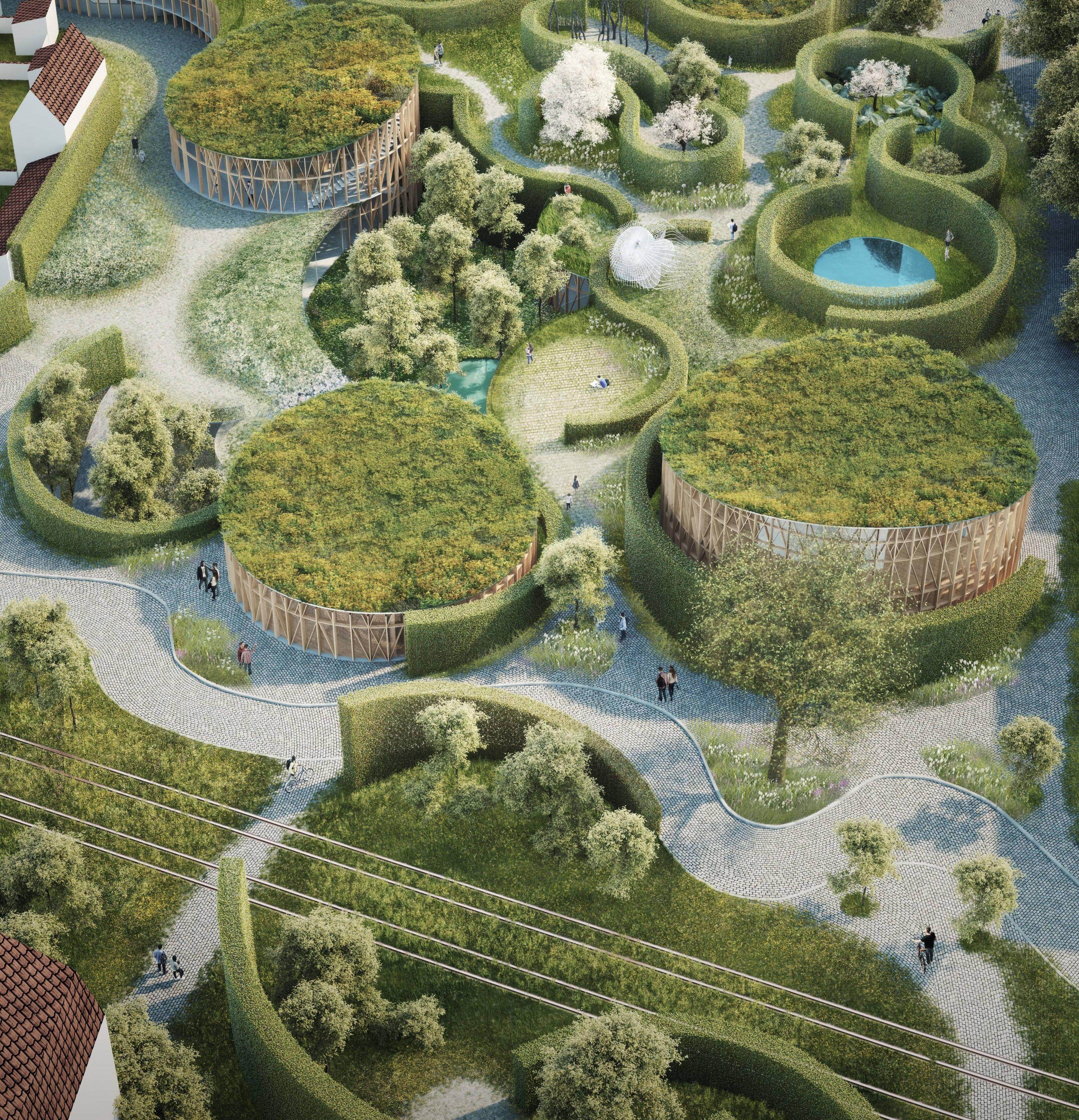


[Single choice question] *

| Very dissatisfied | ○1 | ○2 | ○3 | ○4 | ○5 | ○6 | ○7 | ○8 | ○9 | ○10 | Very satisfied |
| --- | --- | --- | --- | --- | --- | --- | --- | --- | --- | --- | --- |

49. This scene makes me feel uplifted


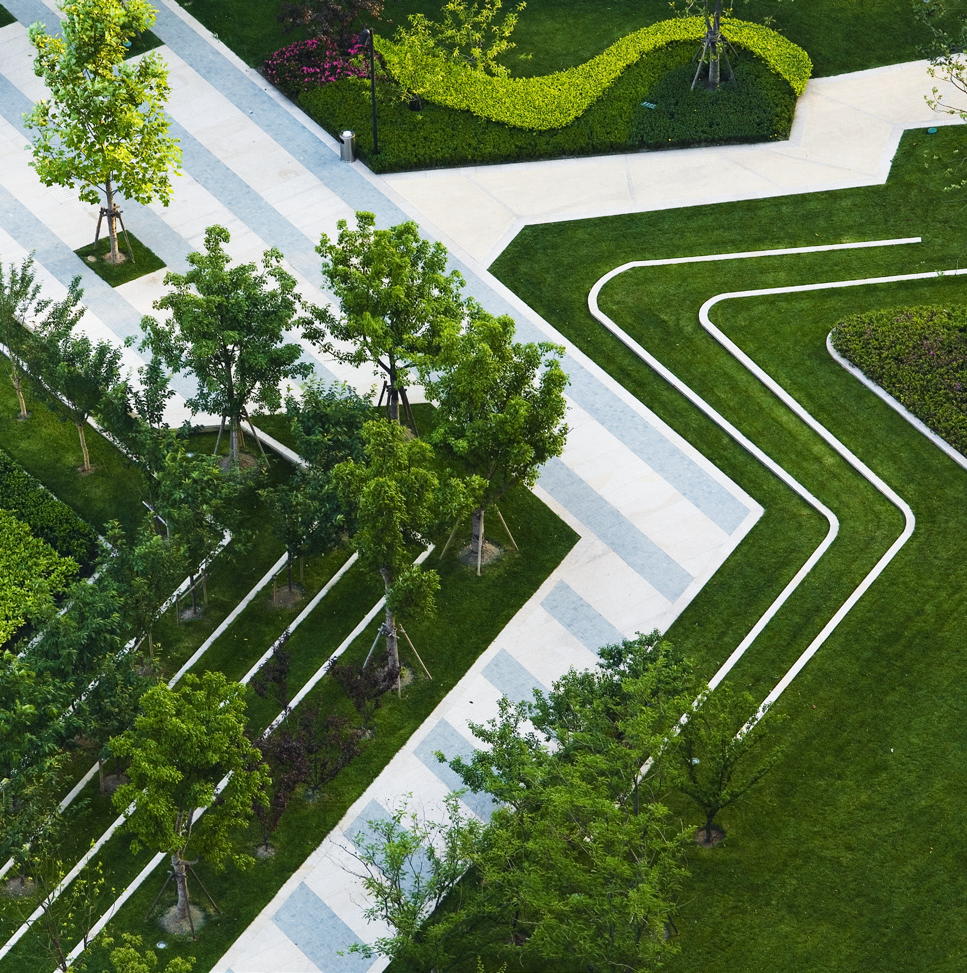


[Single choice question] *

| Very dissatisfied | ○1 | ○2 | ○3 | ○4 | ○5 | ○6 | ○7 | ○8 | ○9 | ○10 | Very satisfied |
| --- | --- | --- | --- | --- | --- | --- | --- | --- | --- | --- | --- |

50. This scene makes me feel relaxed


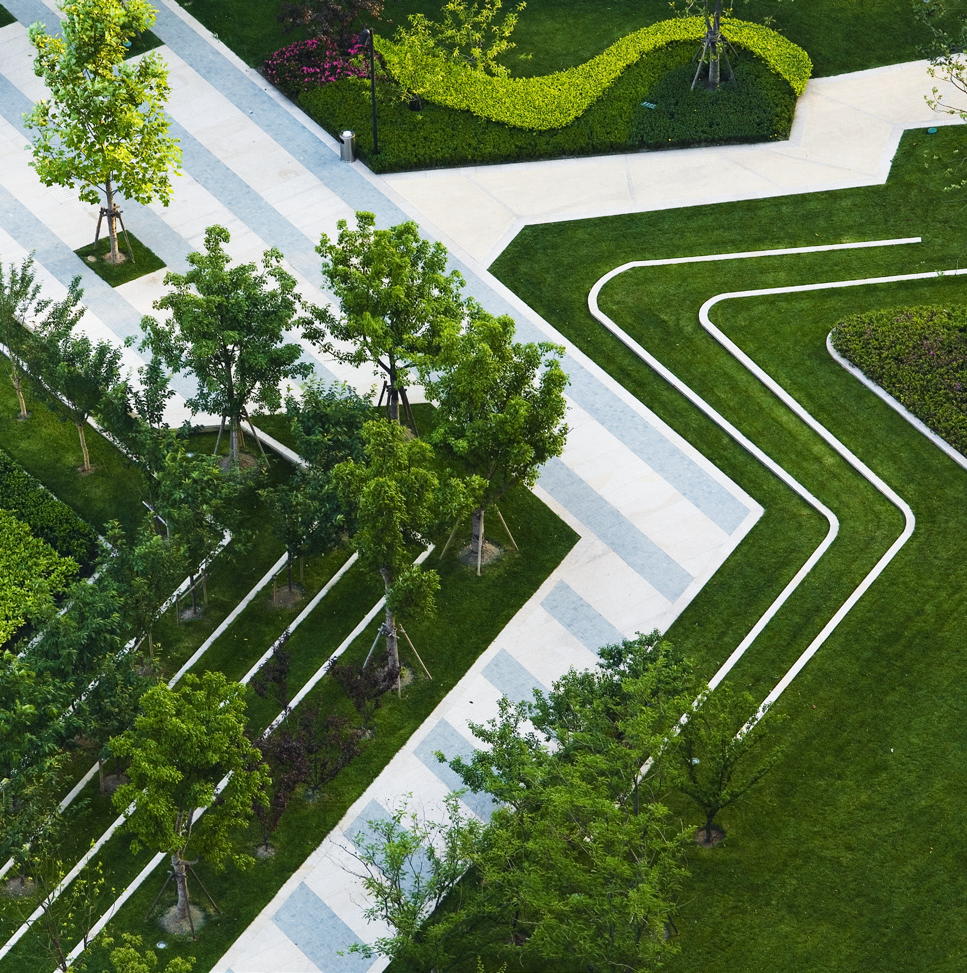


[Single choice question] *

| Very dissatisfied | ○1 | ○2 | ○3 | ○4 | ○5 | ○6 | ○7 | ○8 | ○9 | ○10 | Very satisfied |
| --- | --- | --- | --- | --- | --- | --- | --- | --- | --- | --- | --- |
