# Supplementary material for: From emotional map to design criteria: verification of the correlation between community green space form and emotional health of high-density urban residents
Source: Front Public Health. 2025 Jul 15;13:1617294. doi: 10.3389/fpubh.2025.1617294 (PMC12306530; doi:10.3389/fpubh.2025.1617294)
Supplement: Supplementary file 1 [file Data_Sheet_1.zip › Survey questionnaire content and raw data/The average score.docx]

Appendix A

**Table A1.** The average score of each dimension

| Uplifted Emotion | Average Value | Relaxed Emotion | Average Value |
| --- | --- | --- | --- |
| Green space richness (0%) | 6.33 | Green space richness (0%) | 6.03 |
| Green space richness (25%) | 7.37 | Green space richness (25%) | 6.40 |
| Green space richness (50%) | 6.81 | Green space richness (50%) | 7.43 |
| Green space richness (75%) | 5.76 | Green space richness (75%) | 6.22 |
| Green space richness (100%) | 5.99 | Green space richness (100%) | 6.77 |
| The flower and grass combination ratio (0%) | 5.74 | The flower and grass combination ratio (0%) | 7.07 |
| The flower and grass combination ratio (25%) | 6.00 | The flower and grass combination ratio (25%) | 5.91 |
| The flower and grass combination ratio (50%) | 7.52 | The flower and grass combination ratio (50%) | 6.18 |
| The flower and grass combination ratio (75%) | 6.46 | The flower and grass combination ratio (75%) | 6.47 |
| The flower and grass combination ratio (100%) | 6.89 | The flower and grass combination ratio (100%) | 6.47 |
| Green space connectivity (0%) | 7.24 | Green space connectivity (0%) | 6.29 |
| Green space connectivity (25%) | 6.88 | Green space connectivity (25%) | 7.07 |
| Green space connectivity (50%) | 6.36 | Green space connectivity (50%) | 6.17 |
| Green space connectivity (75%) | 6.88 | Green space connectivity (75%) | 6.86 |
| Green space connectivity (100%) | 5.90 | Green space connectivity (100%) | 6.47 |
| High difference green spaces | 6.31 | High difference green spaces | 6.47 |
| Curved green spaces | 7.31 | Curved green spaces | 6.34 |
| Rectangular green spaces | 6.00 | Rectangular green spaces | 7.28 |
| Circular green spaces | 6.20 | Circular green spaces | 6.53 |
| Broken-line green space | 6.92 | Broken-line green space | 6.89 |

Appendix B

**Table B1.** The impact of different ages of the surveyed population on the score of uplifting emotions

| **Uplifted Emotion** | ＜18 | 18~25 | 26~30 | 31~40 | 41~50 | 51~60 | 60+ |
| --- | --- | --- | --- | --- | --- | --- | --- |
| Green space richness (0%) | 5.97 | 6.33 | 6.69 | 6.15 | 6.68 | 5.96 | 5.69 |
| Green space richness (25%) | 7.53 | 7.33 | 7.59 | 7.25 | 7.2 | 7.39 | 7.31 |
| Green space richness (50%) | 7.13 | 7.07 | 6.92 | 6.71 | 6.75 | 6.32 | 6.62 |
| Green space richness (75%) | 5.69 | 5.98 | 5.86 | 5.7 | 5.71 | 5.46 | 5.54 |
| Green space richness (100%) | 6.5 | 6.46 | 6.06 | 5.78 | 6.07 | 5.36 | 5.85 |
| The flower and grass combination ratio (0%) | 5.41 | 6.2 | 5.95 | 5.61 | 5.88 | 5.32 | 4.62 |
| The flower and grass combination ratio (**25**%) | 6.25 | 6.54 | 6.1 | 5.85 | 6.02 | 5.43 | 5.23 |
| The flower and grass combination ratio (**50**%) | 7.56 | 7.87 | 7.71 | 7.32 | 7.61 | 7.21 | 7.23 |
| The flower and grass combination ratio (**75**%) | 6.91 | 6.5 | 6.56 | 6.37 | 6.73 | 5.86 | 5.62 |
| The flower and grass combination ratio (**100**%) | 7.19 | 7.17 | 7.26 | 6.68 | 6.77 | 6.61 | 5.69 |
| Green space connectivity (0%) | 6.75 | 7.44 | 7.5 | 7.33 | 7.07 | 6.61 | 6.46 |
| Green space connectivity (**25**%) | 6.56 | 6.91 | 7.14 | 6.89 | 6.95 | 6.29 | 6.23 |
| Green space connectivity (**5**0%) | 7.25 | 7.06 | 6.41 | 6.08 | 6.27 | 6 | 5.46 |
| Green space connectivity (**75**%) | 6.13 | 7.17 | 7.16 | 6.93 | 6.75 | 6.39 | 6.23 |
| Green space connectivity (**10**0%) | 6.03 | 5.93 | 6.28 | 5.74 | 5.7 | 5.89 | 5.23 |
| High difference green spaces | 6.72 | 6.37 | 6.65 | 6.07 | 6.54 | 5.79 | 5.31 |
| Curved green spaces | 6.91 | 7.46 | 7.54 | 7.29 | 7.48 | 6.57 | 6.92 |
| Rectangular green spaces | 5.25 | 6.09 | 6.18 | 6.05 | 6.32 | 5.71 | 4.62 |
| Circular green spaces | 6.84 | 6.41 | 6.3 | 6.15 | 5.79 | 5.64 | 6.46 |
| **Broken-line** green space | 6.59 | 7 | 7.21 | 6.81 | 7.14 | 6.54 | 6.08 |

**Table B2.** The impact of different ages of the surveyed population on the score of relaxation emotions

| **Relaxed Emotion** | ＜18 | 18~25 | 26~30 | 31~40 | 41~50 | 51~60 | 60+ |
| --- | --- | --- | --- | --- | --- | --- | --- |
| Green space richness (0%) | 6.19 | 6.09 | 6.32 | 6.02 | 5.88 | 5.57 | 4.85 |
| Green space richness (25%) | 6.72 | 6.22 | 6.78 | 6.28 | 6.38 | 6.14 | 5.31 |
| Green space richness (50%) | 6.91 | 7.11 | 7.62 | 7.6 | 7.54 | 6.71 | 7.23 |
| Green space richness (75%) | 6.5 | 6.07 | 6.51 | 6.09 | 6.45 | 5.61 | 5.46 |
| Green space richness (100%) | 6.88 | 7.17 | 6.99 | 6.65 | 6.64 | 6.36 | 5.85 |
| The flower and grass combination ratio (0%) | 6.97 | 7.2 | 7.16 | 7.2 | 6.7 | 6.54 | 7 |
| The flower and grass combination ratio (25%) | 5.72 | 6.06 | 5.91 | 6 | 6.2 | 5 | 5.31 |
| The flower and grass combination ratio (50%) | 6.25 | 6.63 | 6.39 | 5.96 | 6.21 | 5.61 | 6.15 |
| The flower and grass combination ratio (75%) | 6.81 | 6.72 | 6.54 | 6.32 | 6.77 | 6.14 | 5.08 |
| The flower and grass combination ratio (100%) | 6.47 | 6.8 | 6.75 | 6.4 | 6.64 | 5.29 | 5.38 |
| Green space connectivity (0%) | 6.5 | 6.52 | 6.47 | 6.22 | 6.16 | 6.04 | 5.46 |
| Green space connectivity (**25**%) | 6.78 | 7.3 | 7.37 | 6.82 | 7.36 | 7 | 6.31 |
| Green space connectivity (**5**0%) | 5.88 | 6.57 | 6.49 | 6.04 | 6.07 | 5.79 | 5.38 |
| Green space connectivity (**75**%) | 6.97 | 7.09 | 7.18 | 6.76 | 6.75 | 6.32 | 5.92 |
| Green space connectivity (100%) | 6.84 | 6.93 | 6.31 | 6.4 | 6.59 | 6.14 | 6.23 |
| High difference green spaces | 7.06 | 6.65 | 6.35 | 6.35 | 6.46 | 6.5 | 6.85 |
| Curved green spaces | 6.88 | 6.63 | 6.58 | 6.06 | 6.52 | 5.75 | 5.77 |
| Rectangular green spaces | 6.91 | 7.37 | 7.59 | 7.11 | 7.23 | 7.43 | 7.15 |
| Circular green spaces | 6.53 | 7.04 | 6.86 | 6.2 | 6.66 | 6.5 | 5.08 |
| Broken-line green space | 7.31 | 7.26 | 7.08 | 6.75 | 7.04 | 5.82 | 6.08 |
